# Presence of Human Pathogens of the *Borrelia burgdorferi* sensu lato Complex Shifts the Sequence Read Abundances of Tick Microbiomes in Two German Locations

**DOI:** 10.3390/microorganisms9091814

**Published:** 2021-08-26

**Authors:** Angeline Hoffmann, Thomas Müller, Volker Fingerle, Matthias Noll

**Affiliations:** 1 Department of Applied Sciences, Institute for Bioanalysis, Coburg University of Applied Sciences and Arts, 96450 Coburg, Germany; angeline.hoffmann@hs-coburg.de; 2 Synlab Medical Care Unit, Department of Molecular Biology, Tick Laboratory, Weiden in the Upper Palatinate, 92637 Weiden, Germany; Thomas.Mueller@synlab.com; 3 Bavarian Health and Food Safety Authority (LGL), National Reference Center for Borrelia, 85764 Oberschleißheim, Germany; volker.fingerle@lgl.bayern.de; 4 Bayreuth Center of Ecology and Environmental Research (BayCEER), University of Bayreuth, 95440 Bayreuth, Germany

**Keywords:** bacterial 16S rRNA gene, *Borrelia*, high throughput sequencing, microbiome, network analysis, ticks

## Abstract

The distribution of human Lyme borreliosis (LB) is assumed random in Germany, indicating that the human pathogenic species of the *Borrelia burgdorferi* sensu lato complex (Bb) are similarly distributed as part of the tick microbiome. The aim of this study was to differentiate if the presence of Bb occurs with a defined tick microbiome composition. Furthermore, the effect of location on tick microbiome composition was addressed for two German locations. Therefore, nucleic acid extracts from 82 *Borrelia*-positive and 118 *Borrelia*-negative *Ixodes ricinus* ticks sampled from human hosts in both districts were selected. Nucleic acid extracts were used for human pathogenic Bb species diagnostics based on qPCR and multilocus sequence typing (MLST) and bacterial 16S rRNA gene amplicon sequencing followed by network analyses. As a result, the presence of Bb shifted the sequence read abundances of *Candidatus* Midichloria, *Rickettsia*, *Pseudomonas*, *Staphylococcus*, and *Candidatus* Neoehrlichia and their topological roles in the tick microbiome. Moreover, the location was less important in the tick microbiome composition but shifted significantly sequence read abundances of *Pseudomonas* and *Wolbachia* as well as the topological role of microbial members. Since the presence of human pathogenic Bb species with other tick-associated pathogens varies regionally, we suggest that a bacterial 16S rRNA gene-based microbiome survey should be implemented in the routine diagnostics for both tick and host if human pathogenic species of Bb were detected. This diagnostic extension will help to optimize therapeutic approaches against Bb infection and co-occurring pathogens.

## 1. Introduction

Ticks are obligate blood-sucking ectoparasites with diverse animals and humans as hosts. Therefore, *Ixodes* serve as vectors for tick-borne pathogens (TBPs), including species of the genera *Borrelia*, *Rickettsia*, or *Babesia* [1,2], which can be transmitted from and/or to their hosts. In Germany, *Ixodes ricinus* is the most common tick species and was linked to 13,361 cases of Lyme borreliosis (LB) in 2018 without an identifiable distribution pattern [3,4,5].

LB is a multisystemic infectious disease with the highest incidence in Europe and North American, caused by human pathogenic bacterial species belonging to the *Borrelia burgdorferi* s. l. complex (Bb). Within the at least 20 genospecies comprising Bb, six are assured human pathogenic species, namely: *B. burgdorferi* s. s., *B. garinii*, *B. afzelii*, *B. spielmanii*, *B. mayonii*, and *B. bavariensis* [6]. Tick material, cerebrospinal fluids (CSF), skin, or blood of the hosts are used to analyze these human pathogenic Bb species. To date, PCR is the state-of-the-art technique to detect human pathogenic species of Bb from tick specimens directly with human pathogenic Bb-specific primer sets [7]. However, false positive or false negative PCR results can occur [8,9].

In addition to Bb and other TBPs, ticks also harbor many other microorganisms that are non-pathogenic. When considering this microbial community as a whole, it is generally referred to as the tick microbiome. Within this microbial community, non-pathogenic bacteria such as endosymbionts, commensals, or mutualistic bacteria are also found. The pathways to acquire a microbiome for ticks are manifold [10,11], and the presence and advantageous functions for the host are similarly manifold and are located in various sites of the tick habitats. For example, potential tick-borne microorganisms are present as saprophytes and plant-promoting commensals in soil and plants, or a part as opportunistic pathogens in hosts. Ticks can aquire them through microbial contamination during blood meals and from their environmental habitats [10,12,13].

To explore the bacterial microbiome in tick material, bacterial 16S rRNA gene amplicon sequencing is commonly applied [1,14,15]. Moreover, the use of high throughput sequencing technologies has broadened our understanding of which microbial community compositions can be found in ticks [15]. However, as the tick microbiome is composed only of few distinct genera (*Rickettsia*, *Spiroplasma*, *Pseudomonas*, *Coxiella*, and *Mycobacterium*), which are found in a wide range of tick species [16,17,18], a spontaneous inoculation of any ubiquitous bacteria associated with soil, plants, skin, and blood to the tick host is less likely [19]. Therefore, a combination of a controlled transfer of bacteria within a tick community, its hosts, and invasive bacterial taxa into the microbiome were proposed [15,20]. The female tick plays a crucial role in the microbiome composition by transferring beneficial microbes, important endosymbionts, but also pathogens to the eggs or larvae by vertical transmission [19,21].

Additional information, such as the location of tick collection, can be added to the dataset to identify location-(in)dependent correlations between TBPs and other bacteria. Such information may provide an even better understanding of bacterial composition in the microbiome. In addition, this knowledge may be used as a new diagnostic approach to avoid further false positive or negative results [19,20,22]. Pollet et al. summarised in a review the temporal and spatial scales that may influence tick microbiome composition and found that ticks, and most likely their microbiome, rely on vertebrate movement to prevail as a meta-population [20]. This spatial scale implies that location is a driver of tick microbiome variability. Additionally reviewed that regional conditions of tick reservoirs and habitats are key determinants of bacterial colonization and shifts in the tick microbiome, including tick-associated human pathogens [20,23].

These tick microbiome surveys are very benefical to understand which bacterial community composition can be found, including TBPs, and allow the opportunity to analyze if and how TBPs and non-pathogenic bacteria interact with each other. However, a comprehensive study of whether or/and how human pathogenic Bb species and the location affect the overall tick microbiome composition together and whether the presence of human pathogenic Bb species negates or weakens the already established location dependency is not addressed so far to the best of our knowledge.

Therefore, this study address three a priori hypotheses. (I) the presence of human pathogenic species of the Bb in ticks cause a shift in the composition of the bacterial tick microbiome compared to those bacterial tick microbiomes without human pathogenic species of the Bb, (II) if the occurrence of LB exists without an identifiable distribution pattern in Germany, then the bacterial tick microbiome, including human pathogenic Bb species is also location independent, and finally (III) whether, or to what extent, the location and/or the presence of human pathogenic Bb species has an effect on the co-occurrence of different bacterial community members with their topological role in the bacterial tick microbiome. Therefore, we used 200 tick nucleic acid extracts from two German locations Esslingen (Baden-Wuerttemberg) and Weiden (Bavaria) that were prior screened by the *ospA* gene and further MLST specific PCR assays [24] for the presence of human pathogenic Bb species (EN and EP for *Borrelia*-negative and *Borrelia*-positive extracts from Esslingen and WN and WP for *Borrelia*-negative and *Borrelia*-positive extracts from Weiden). Thereafter, the 200 bacterial tick microbiomes were assessed by bacterial 16S rRNA gene amplicon high throughput sequencing. Finally, the bacterial tick microbiomes were compared, and co-occurrence networks were calculated.

## 2. Materials and Methods

### 2.1. Nucleic Acid Extracts of Ticks and Metadata Management

For the analysis of TBPs, especially human pathogenic species of Bb, 3863 ticks were sent in total to the accredited tick laboratory of Synlab Medical Care Centre (MVZ) Weiden from October 2017 to April 2019, by doctors of or directly by the private person (human host). In addition, data of patient or private person, as name, address with the postal code, and date of submission were deposited for each tick sent to the laboratory. Then the nucleic acid extraction, as explained earlier [14], was carried out, and a real-time TaqMan PCR tested the presence of human pathogenic Bb species with the primer pair (5′-AATATTTATTGGGAATAGGTCTAA-3′ and 5′-CACCAGGCAAATCTACTGA-3′) and probe (tm-FA TTAATAGCATGYAAGCAAAATGTTAGCA) as outlined earlier [7]. Most of the prior screened nucleic acid extracts of ticks by Synlab MVZ were provided to our institute and were stored at −20 °C until further investigation.

To generate morphological insights of ticks sent to Synlab MVZ, ticks were randomly examined prior to nucleic acid extraction. The metadata species, stage, and conditions (flat/engorged) of ticks were documented [25]. While the majority was identified as *I. ricinus* (79.3%), 6.2% were identified as *Ixodes hexagonus*, 1.0% as *Dermacentor reticulatus*, and 0.5% as *Argas reflexus*. 13.5% could not be determined due to their state when removed from the host. Only *I. ricinus* were further analyzed, from which 78.2% were nymphs, and 7.2% were adults. 91.7% of adult *I. ricinus* were not yet engorged (flat), and 8.3% were already engorged. However, since we already received extracted DNA from ticks, this information could unfortunately not be considered in the study.

To compare a high amount of tick extracts for the analysis of the hypothesis, the regional postal codes of nucleic acid extracts from ticks available for the study from 2018 were mapped on a map of Germany using Rstudio and the packages sf, tmap, tmaptools, dplyr, and ggplot [26,27,28,29,30] (Supplementary Figure S1). Based on the resulting map, Neustadt an der Waldnaab with the independent city of Weiden in der Oberpfalz (Upper Palatinate, Bavaria) and Esslingen (Stuttgart, Baden-Wuerttemberg) were selected, as both locations offered a high number of *Borrelia*-positive and *Borrelia*-negative tick extracts for detailed microbiome analyses. Further, Esslingen, with its 29 natural reserves and an area of about 2320 ha (3.62% of the total county area), offers several protected areas for endangered and protected plants and wildlife in contrast to Weiden with only 13 natural reserves with a total of 787 ha (0.55% of the total county area) [31,32]. Moreover, the location of Esslingen offers a slightly warmer (average temperature: 9.2 °C [33] and a more heterogeneous, rather fragmented sylvatic biotope with pastures, steep slopes, heaths, compensation areas, mainly deciduous forests, meadow floodplains, wetlands, and rocks. In contrast, Weiden offers a rather cooler (average temperature: 7.8 °C [34], more natural and rather less fragmented sylvatic environment with many bogs, wet lowlands with peat deposits, unwooded ridges, and many inaccessible areas with granite and basalt.

In addition, the nucleic acid extracts with the human pathogenic Bb species findings were confirmed by a multilocus sequence typing (MLST) with eight housekeeping genes and additionally the *p41* gene [35,36,37]. Therefore, the nucleic acid extracts from the ticks were grouped to Esslingen *Borrelia*-positive tick extracts (EP, *n* = 38), Esslingen *Borrelia*-negative tick extracts (EN, *n* = 62), Weiden *Borrelia*-positive tick extracts (WP, *n* = 44), and Weiden *Borrelia*-negative tick extracts (WN, *n* = 56).

### 2.2. PCR Amplification for Tick Samples and Bacterial 16S rRNA Gene Sequencing

The V3-V4 region of the bacterial 16S rRNA gene (primers: 341F: 5′-CCTACGGGNGGCWGCAG-3′ and 785R: 5′-GACTACHVGGGTATCTAATCC-3′ [38] of nucleic acid extracts from each tick were amplified by PCR. 13.5 µL UltraPure™ DNase/RNase free distilled water (Thermo Fisher Scientific, Waltham, MA, USA), 10 µL 5× HF buffer (Thermo Fisher Scientific), 2 µL forward (5 µM) and 10 µL reverse primer (5 µM), 1 µL dNTP Mix [10 mM] (Thermo Fisher Scientific), 0.5 µL Phusion high-fidelity polymerase (Thermo Fisher Scientific), 10 µL 5× PCR-Enhancer (Biozym Scientific GmbH, Hessisch Oldendorf, Germany) and 3 µL (5 to 75 ng genomic DNA) of tick nucleic acid extract were applied for each PCR reaction. For a negative control UltraPure™ water (Thermo Fisher Scientific) and for positive control, a nucleic acid extract of *Escherichia coli* (DSM 423) were used. PCR thermal profile was an initial denaturation at 98 °C for 2 min followed by 30 cycles of denaturation at 98 °C for 5 s, annealing at 57.4 °C for 30 s, extension 72 °C for 30 s, and the final elongation at 72 °C for 10 min in a T100™ Thermal Cycler (Bio-Rad Laboratories GmbH, Feldkirchen, Germany). PCR products were checked by a 1.5% agarose gel, stained with ethidium bromide, and visualized by UV light (Genoplex, VWR International GmbH, Darmstadt, Germany).

Thereafter PCR products were sent to LGC Genomics GmbH (Berlin, Germany), and bacterial 16S rRNA gene amplicon sequencing using PCR primers as sequencing primers, raw bioinformatic data pre-processing, quality checking, and sequence pre-processing was carried out as reported recently [14]. As a result of these analyses, an operational taxonomic unit (OTU) table was created. A total of 17,696,484 sequences were obtained after filtering, which corresponded to 1944 bacterial OTUs, respectively (Supplementary Table S1). All singletons were filtered from the OTU table, and OTUs were summarised on the genus level (Supplementary Table S2). In addition, the reads of the OTU table were converted into binary and relative sequence read abundances [39].

### 2.3. Statistics

Correspondence analysis (CA) was performed with the converted OTU table (binary and relative sequence read abundances) summarized on location and assignment to human pathogenic Bb species presence (EN, EP, WN, WP) using RStudio and the package FactoMineR [40] to get the first insight into differences in the bacterial community compositions. Cluster analyses were carried out with a Euclidian distance according to the ward.D2 method between the composition of tick microbiomes (first two dimensions of CA) by using the functions dist and hclust in the package stats [41] and visualized by the package dendextend [42]. The ten most abundant genera reflected 72 to 83% (as a subset of *Borrelia* findings and location; WN, EN, WP, and EP) of relative abundances, which were illustrated using Origin 2017 (OriginLab Corporation, Northampton, MA, USA) to compare the bacterial community composition between groups. Significant effects (*p* < 0.05) between the relative frequencies of the ten most common genera between groups (EN, EP, WN, WP) were calculated using Kruskal–Wallis ANOVA by using Origin 2017 (OriginLab Corporation) to exclude the apparent significance of bacterial genera.

### 2.4. Analysis of Microbial Networks

The network analyses were performed using CoNet App, an integrated app in Cytoscape version 3.7.2 [43,44]. As outlined above, the networks were calculated separately for each group (EN, EP, WN, and WP), each with an individual OTU table with relative abundances. Then, Pearson, Spearman correlation, Bray Curtis, and Kullback–Leibler dissimilarity and Hellinger distance were combined to create a robust network. 3000 edges (top and bottom) were chosen as the threshold for network calculation [45,46]. Finally, only edges were presented that were supported by at least three out of these five statistical methods. Finally, the statistical evaluation of the obtained networks was achieved by calculating 100 random networks using the permutation and bootstrap method [47]. Topological roles of each node in all four networks were defined as previously described [48].

Furthermore, the average closeness centrality and the average clustering coefficient of all four networks were determined. The closeness centrality of a node is a measure of centrality in a network and can be interpreted as measuring the distance between one node to another in the network [49]. The clustering coefficient, in turn, provides a measure of the degree of interconnectivity in a node’s neighborhood [50].

## 3. Results

### 3.1. Presence of Human Pathogenic Bb Species Affected the Tick Microbiome Composition

**Figure 1:**
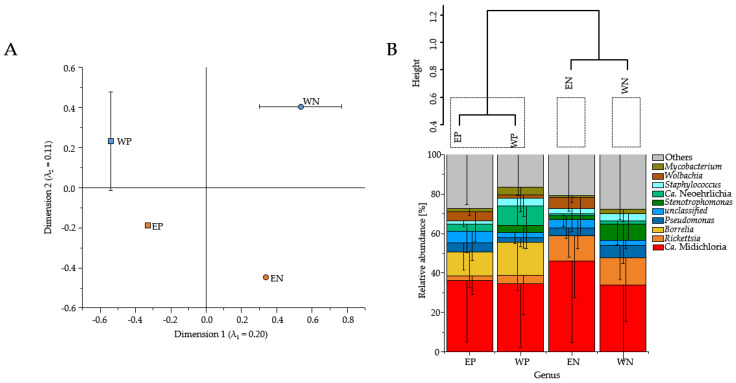
Correspondence analysis of bacterial community compositions (**A**), Euclidean distance matrix based on ward.D2 method (B above), and bacterial community composition ((**B**) below) of *Borrelia*-negative ticks retrieved from Esslingen (EN; *n* = 62; orange circle) or Weiden (WN; *n* = 56, blue circle) and *Borrelia*-positive ticks retrieved from Esslingen (EP; *n* = 38; orange square) or Weiden (WP; *n* = 44; blue square). Bacterial community composition is based on genus level with relative abundance data. The eigenvalues of both axes and standard errors are shown (**A**). For clusters, heights of 0.6 were chosen and denoted in dashed boxes ((**B**) above). Bacterial community composition of the ten most abundant genera are shown, and other genera are summarized as “others”. For colors and patterns, see figure legend.

The presence of human pathogenic Bb species shifted the bacterial community composition in both locations (Figure 1A); EP and WP were clustered more closely than EN and WN (Figure 1B). Moreover, the Euclidean distances showed that the presence of human pathogenic Bb species (EP and WP) coincided in one cluster, while the absence of human pathogenic Bb species (EN and WN) lead to distinct clusters independently if binary or relative abundances were used (see Figure 1B and Supplementary Figures S2 and S3).

**Figure 2:**
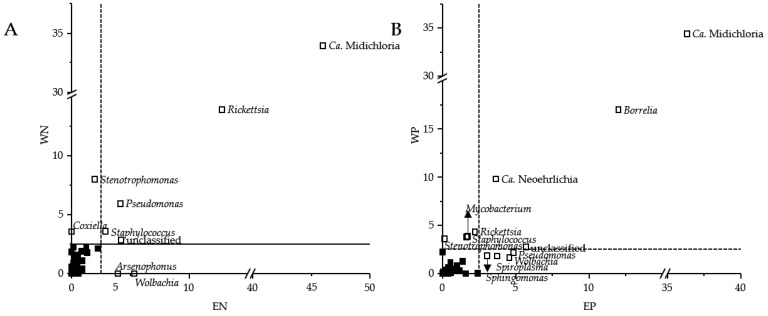
Differences in the sequence read abundances of bacterial genera obtained from tick-borne *Borrelia*-negative (**A**) (EN, *n* = 62; WN, *n* = 56) and *Borrelia*-positive bacterial community compositions (**B**) (EP; *n* = 38; WP; *n* = 44). Only bacterial genera were shown with a mean relative abundance of at least 2.5% in Esslingen (E) or Weiden (W).

The shift in the bacterial community composition by the presence of human pathogenic Bb species was caused by higher sequence read abundances of *Candidatus* (*Ca*.) Midichloria, *Rickettsia*, *Pseudomonas*, *Staphylococcus* and an unclassified bacterial OTU in *Borrelia*-negative tick extracts and Ca. Midichloria, *Borrelia*, Ca. Neoehrlichia, and another bacterial unclassified OTU in *Borrelia*-positive tick extracts (Figure 2). While Ca. Midichloria was still present in each tick-borne bacterial community composition (EP, WP, EN and WN) with approximately the similar high sequence read abundances, there was a noticeable reduction of the genus *Rickettsia* in the *Borrelia*-positive tick extracts (EP: 2.2%, WP: 4.3%, EN: 12.8% and WN: 13.9%) (Figure 1B and Figure 2). Moreover, Ca. Neoehrlichia showed higher sequence read abundances in *Borrelia*-positive tick extracts (EP, 3.7%; WP, 9.8%) than *Borrelia*-negative tick extracts (EN, 0.9%; WN, 1.9%). Abundant bacterial genera differed in each tick-borne bacterial community composition (EP, WP, EN and WN). These included the genera *Stenotrophomonas* for WN and WP, *Coxiella* for WN, *Arsenophonus* and *Wolbachia* for EN, *Rickettsia*, *Staphylococcus* and *Mycobacterium* for WP and *Sphingomonas*, *Spiroplasma*, *Wolbachia* as well as *Pseudomonas* for EP (Figure 2). The bacterial genera *Mycobacterium* (*p* = 1.35 × 10^−3^) as well as *Borrelia* (*p* = 2.02 × 10^−41^) and Ca. Neoehrlichia (*p* = 9.19 × 10^−3^) were significantly more abundant in *Borrelia*-positive compared to *Borrelia*-negative tick extracts.

The richness (*p* = 1.62 × 10^−3^) and Shannon index (*p* = 2.58 × 10^−5^) were significantly lower in *Borrelia*-negative than *Borrelia*-positive tick extracts, as well as Pielou’s evenness (*p* = 4.74 × 10^−42^) were significantly higher in *Borrelia*-positive compared to *Borrelia*-negative tick extracts. However, these indices were non-significantly different between both locations (Table S3).

### 3.2. Sequence Read Abundances of Wolbachia and Pseudomonas Differed between Locations 

**Figure 3:**
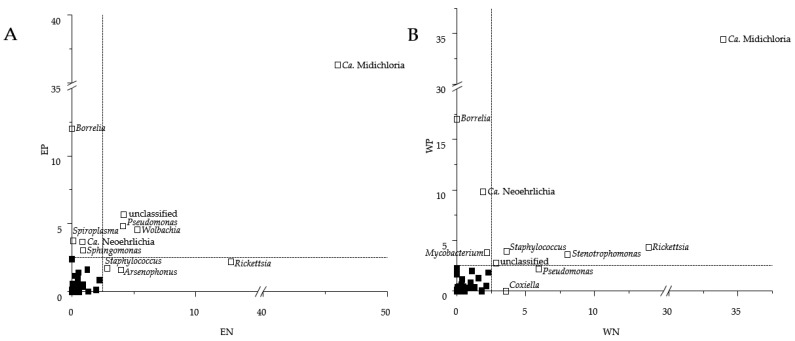
Differences in the sequence read abundances of bacterial genera obtained from tick-borne bacterial community compositions from Esslingen (**A**) (EN, *Borrelia*-negative, *n* = 62; EP, *Borrelia*-positive, *n* = 38) and Weiden (**B**) (WN, *Borrelia*-negative, *n* = 56; WP, *Borrelia*-positive; *n* = 44). Only bacterial genera were shown with a mean relative abundance of at least 2.5% in Esslingen (E) or Weiden (W).

The bacterial community compositions of ticks retrieved from Esslingen (EN and EP) differed from those from Weiden (WN and WP) (see Figure 1). The differences in the bacterial community composition between Esslingen and Weiden were based on the sequence read abundances of Ca. Midichloria, an unclassified bacterial OTU, *Pseudomonas*, and *Wolbachia* in ticks retrieved from Esslingen (EN vs. EP) and of Ca. Midichloria, *Rickettsia*, *Staphylococcus*, *Stenotrophomonas*, and the same unclassified OTU in ticks retrieved from Weiden (WN vs. WP) (Figure 1B and Figure 3). Furthermore, EP and WP were governed by the bacterial genera *Borrelia* and Ca. Neoehrlichia, while *Spiroplasma* and *Sphingomonas* specifically dominated EP and *Mycobacterium* WP. On the other hand, *Staphylococcus*, *Arsenophonus*, and *Rickettsia* were more frequently abundant for EN, and *Coxiella* and *Pseudomonas* for WN. The sequence read abundances of the bacterial genera *Wolbachia* (*p* = 1.01 × 10^−3^), and *Pseudomonas* (*p* = 2.07 × 10^−2^) were significantly higher from ticks retrieved from Esslingen compared to those from Weiden. However, the bacterial genera *Stenotrophomonas* (*p* = 0.085), *Staphylococcus* (*p* = 0.369), as well as *Rickettsia* (*p* = 0.730) were non-significantly higher in ticks obtained from Weiden compared to those from Esslingen.

### 3.3. Presence of Human Pathogenic Bb Species Shifted Co-Occurrence in the Tick Microbiome

**Figure 4:**
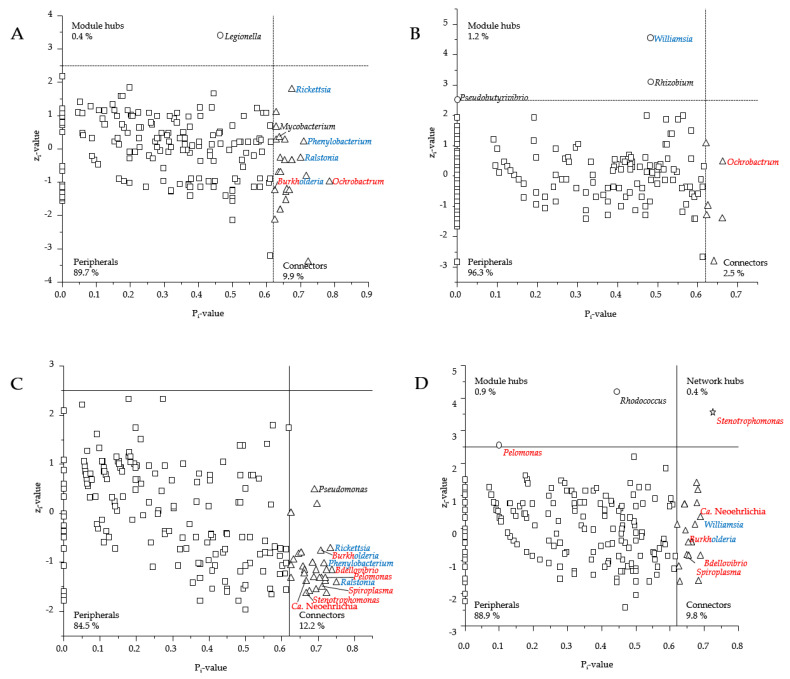
Topological roles of each node (bacterial genus) of a microbial network analyses for EN ((**A**); *n* = 62), EP ((**B**); *n* = 38), WN ((**C**); *n* = 56) and WP ((**D**); *n* = 44) according to their zi and Pi values. All nodes of the networks were categorized into one of four groups (peripherals, connectors, network and module hubs) according to their z_i_ and P_i_ values, as Olesen et al. suggested [48]. Module hubs are marked as a circle, network hubs as a star, peripherals as square, and connectors as a triangle. The blue labeled genera occurred in the other network of the same locus (EN and EP or WN and WP) with the same or changed topological role (blue), while the red labeled genera occurred in the network of the other locus (EN and WN or EP and WP) with the same or changed topological role. An overview of the co-occurrence patterns of all nodes can be found in the appendix (Supplementary Table S2).

The co-occurrence networks differed in their number of nodes and edges between EN, EP, WN, and WP, and the networks derived from EN and WN were more complex than those from EP and WP based on the number of clusters (Supplementary Table S4). Networks derived from EN and WN showed a higher clustering coefficient in comparison to EP and WP. Regardless, a similar closeness centrality was found for all four networks. Networks derived from EN and WN had a higher proportion of connectors (9.9% and 12.2%) compared to EP (2.5%), while the number of module hubs was two and three times higher for EP and WP than for EN and WN, respectively (Figure 4). Overall, one network hub (*Stenotrophomonas* in WP) was identified (see Figure 4D).

The network analyses showed that most bacterial genera co-occurred in the same patterns independently from the presence of human pathogenic Bb species or location, which was reflected in their respective topological role (Figure 4). However, the bacterial genera *Ralstonia*, *Clostridium*-sensu-stricto-19, *Rickettsia*, and *Phenylobacterium* were identified as connectors in the *Borrelia*-negative networks of EN and WN (see Figure 4A,C). In contrast, no such observation was obtained in *Borrelia*-positive networks of EP and WP. However, the topological role of *Williamsia* from a module hub in EP was found to be a connector in WP network (see Figure 4B,D).

Some bacterial genera obtained the same topological role as connectors in more than one network, such as *Ochobactrum* for Esslingen networks (EN, EP) and *Spiroplasma*, *Bdellovibrio*, *Burkholderia* as well as Ca. Neoehrlichia for Weiden networks (WN, WP). Furthermore, the topological roles of *Pelomonas* and *Stenotrophomonas* were classified as connectors in WN but were module hub and network hub in WP, respectively (see Figure 4C,D).

## 4. Discussion

### 4.1. Presence of Human Pathogenic Bb Species Shifted Sequence Read Abundances of Bacterial Genera instead of Bacterial Community Composition

*Borrelia*-negative and *Borrelia*-positive microbiomes differed significantly as they revealed distinct clusters regardless of location (Figure 1A). The presence of human pathogenic Bb species in the tick microbiome was only weakly associated with shifts in the bacterial community composition. However, it was highly associated with shifts in sequence read abundances of the bacterial genera in *Borrelia*-negative and *Borrelia*-positive tick microbiomes, as revealed when all microbiomes were analyzed with excluding *Borrelia* sequences (Supplementary Figure S4). Our findings of lower versus higher sequence read abundances of bacterial taxa in the presence of human pathogenic Bb species in the microbiome of *I. ricinus* were in line with results of other tick species (*Ixodes scapularis* (laboratory-reared and field-collected) and *Amblyomma americanum*) [16,18,51]. These findings indicate that not only the presence of human pathogenic Bb species shifted the tick-borne community composition but also the interaction with human pathogenic Bb species and interaction patterns within the tick-specific microbial community.

Our results showed that the sequence read abundances of Ca. Neoehrlichia and *Mycobacterium* were significantly higher in the *Borrelia*-positive microbiomes (Figure 1B and Figure 2A,B), suggesting a clinical threat of co-infection with *Borrelia*, as *Neoehrlichia mikurensis* and *B. afzelii* have already been described in Romania [52]. The prevalence of *N. mikurensis* (8.4% [53]) and *B. afzelii* (30.5% [54]) can be estimated as very high in Germany, indicating that these species are common in tick microbiomes. The explanation for this positive association between both species is that *N. mikurensis* is a tick-borne pathogen that uses rodents as a reservoir host [53]. Similarly, the Bb contains numerous rodent-adapted genospecies such as *B. afzelii* and *B. bavariensis* [55]. Ticks that take their larval and/or nymphal blood meals from rodent hosts are likely to be infected with these two pathogens.

In contrast, ticks that feed on other vertebrate hosts, such as deer, incompetent hosts for *B. afzelii* and *B. bavariensis*, will not be infected with these pathogens. Therefore, researchers should consider whether the strong positive association is present when analyzing the ticks, excluding morphological data (tick species, life stage, sex, engorged/flat) or whether it reflects the fact that the ticks have fed on different vertebrate hosts that differ in their competence to maintain tick-borne pathogens. In contrast to Ca. Neoehrlichia, *Mycobacterium* are widely distributed in various environments such as soil, water, human and animal hosts [56]. The majority of tick-borne microbiome analyses based on 16S rRNA gene surveys have frequently reported worldwide members of the genus *Mycobacterium* [18,57,58]. Therefore, these members were also common in tick microbiomes. For example, *Mycobacterium smegmatis* is known to possess specific regulators capable of generating novel survival-related tick morphotypes during *B. burgdorferi* migration in nymphs [57,58].

The tick microbiome was strongly dominated only by few bacterial genera (Figure 1B). The majority of genera (*Rickettsia*, *Wolbachia*, *Coxiella*, *Spiroplasma* and *Arsenophonus*) were classified as endosymbionts in one of the following hard ticks *Ixodes pacificus*, *Ixodes angustus*, *Dermacentor variabilis*, *Dermacentor occidentalis*, *Dermacentor albipictus,* and *Haemaphysalis leporispalustris* [17]. Such endosymbionts play an important role in the physiology of the tick and are believed to be essential for its survival, for instance, in vitamin synthesis [21]. Moreover, these endosymbiotic genera were described to interact with co-present human pathogenic bacterial taxa (*Anaplasma*, *Borrelia*) of the tick microbiome [17]. In addition, the colonization of *Anaplasma phagocytophilum* in the microbiome of *I. scapularis* caused a decreasing occurrence of members of the genera *Rickettsia* and *Enterococcus* but an increase of *Pseudomonas* [59,60]. Our study showed that the sequence read abundances of the genus *Rickettsia* were particularly higher in *Borrelia*-negative than *Borrelia*-positive microbiomes (Figure 1B and Figure 2A,B), indicating that there is a correlation between *Borrelia* and *Rickettsia* in the tick microbiome. Kowalec et al. found a significant positive correlation between the genus *Rickettsia* and Spirochaetes in *I. ricinus* nymphs [61]. However, the pathogens of these genera occur in different inner organs of the tick. Therefore, it remains to be clarified how these pathogens can interact with each other and what advantages the pathogens may gain. Next to *Rickettsia*, *Pseudomonas* and *Staphylococcus* revealed high sequence read abundances in the *Borrelia*-negative but not *Borrelia*-positive microbiomes (Figure 2A), which is in line with previous results from *I. scapularis* [18]. Moreover, both genera were described to inhibit *Borrelia* transmission next to other microbes of the microbiome [18]. Another study supports our findings of abundant *Pseudomonas* in *Borrelia*-negative microbiomes as they carry a Type VI secretion system, supporting antagonistic interactions with *Borrelia* [62].

Furthermore, the genus *Staphylococcus* was also characterized to interact antagonistically with *Borrelia* [18]. In addition, the infection of *I. scapularis* with *A. phagocytophilum* altered sequence read abundances in the tick-borne microbiome and the ability of *Staphylococcus* to form biofilms in the tick gut, which was assigned to be beneficial to inhibit the transmission of *A. phagocytophilum* [59]. However, the role of other members of the tick microbiome on the *Borrelia* colonization or their presence has not yet been sufficiently investigated [21].

### 4.2. Is the Location Important for the Composition of the Tick Microbiome?

As the LB is almost equally distributed in Germany [5], we hypothesized that the bacterial tick microbiome, including the *Borrelia* findings, is location-independent. However, this hypothesis was rebutted as we found significant differences in the sequence read abundances of *Wolbachia* between both locations (Figure 3A,B). Members of the genus *Wolbachia* were described as mutualistic and capable of infecting mainly arthropods, including insects, and nematodes. Members of the genus *Wolbachia* can be transmitted by endoparasitoid wasps *Ixodiphagus hookeri* to the ticks by oviposition [63], while ticks without contact to *I. hookeri* were free from *Wolbachia* [63,64]. However, such transfer by *I. hookeri* is expected to be at least 20% in natural populations of *I. ricinus* in France [63], and similar findings can be expected in this study for *I. ricinus* as *I. hookeri* is domestic in Germany.

As already mentioned, Pollet et al. summarised in a review the temporal and spatial scales that affect tick microbiome compositions and found that ticks and most likely their microbiome rely on vertebrate movement to prevail as a meta-population [20]. This implies that location is a driver of variability in the tick microbiome, and regional composition or structure of vegetation (more natural, less fragmented sylvatic environment, or a more fragmented sylvatic environment) is essential for ticks and their hosts [20]. These studies concluded that landscape topography, climatic conditions, and vegetation are strongly associated with tick and host development [20,22,65,66,67], indicating why the tick microbiome composition between Esslingen and Weiden differed. Thus, an explanation of the significant differences in sequence read abundances of *Pseudomonas* and *Wolbachia* in Esslingen (Figure 3A) is the different regional conditions as well as the geographical separation from each other with different flora and fauna. Therefore, both locations differed in their connectivity patterns of the landscape, which in turn influences the presence or absence of hosts (variable host composition). Secondly, the spatial diversity within a location (microclimate) thus affecting the tick density, leading to additional variability of the tick microbiome [20,23,51]. Finally, regional conditions contribute to microbial interaction patterns, shifting the acquisition/refusal of additional members into the tick microbiome (see Section 4.1), thereby playing a crucial role in the composition and sequence read abundances of the tick microbiome [65].

However, besides regional structuration, many other tick-associated factors influence and cause variation in the tick microbiome [66,67]. First and foremost, the female tick plays a crucial role in the composition of the microbiome by transferring beneficial microbes, important endosymbionts, but also pathogens to the eggs or larvae by vertical transmission [19,21]. Furthermore, tick species, life stage and sex, nutritional status (fed/unfed), and host as the source of blood meal play key roles in the diversity of the tick microbiome [10,11,19,65,66,67]. These factors contribute to a regionally defined tick microbiome and more research is needed to unravel the importance of each factor on the composition and abundance of the tick microbiome. Unfortunately, there was a lack of such information in our study, as we received already extracted tick nucleic acids. Therefore, it would be advantageous to repeat and support our hypotheses with complete morphological information, as life stage, sex or status of engorgement of each tick.

### 4.3. How Does the Presence of Human Pathogenic Bb Species Shift the Bacterial Networks?

High bacterial variability within the microbiomes of diverse tick species was found [17,18,68]. Based on co-occurrence networks of these microbiomes, we were interested in elucidating location and *Borrelia*-finding specific topological roles of the respective bacterial members. Strikingly, none of the four networks was characterized by particularly high numbers of mutual exclusion (Supplementary Figures S5–S8), suggesting that the tick microbiomes were not strongly characterized by microbial competition, which is in line with previous findings [19]. Furthermore, large nodes were absent, indicating a robust and resilient co-occurrence network as removing one node would only slightly affect the connectivity of the others. Therefore, these network topologies indicate the lack of trophic dependences or competition between co-occurring bacterial genera, which is in line with previous findings [68]. The average cluster coefficient of all four networks was similar (EN: 0.69 ± 0.27, EP: 0.57 ± 0.29, WN: 0.70 ± 0.27, WP: 0.58 ± 0.28), which contradicts the drawbacks of the “small world” theory [69] and supports the findings mentioned earlier [70].

The majority of negative interactions (mutual exclusion) were found by members of the genus *Rickettsia* (Supplementary Figures S13–S16), which is in line with previous findings of the tick microbiome of *I. pacificus* [19]. However, to date, it is still unclear whether *Rickettsia* is directly displacing or promoting other microorganisms or is independently present while other bacteria were decreasing over time in the tick microbiome [17,19]. Members of the genus *Rickettsia* played particularly in the *Borrelia*-negative microbiomes an important topological role as a connector (Figure 4A,C). Connectors are composed of generalists, as they have little to no specialization in their network behavior and can use many different resources [48]. Furthermore, the set of connectors in a network can be used to estimate modularity as they relate to each other [48]. Thus, modularity increases with the specificity of the connections, as shown by the high number of modules (clusters, Supplementary Figures S9–S12), as well as the high proportion of connectors in the *Borrelia*-negative microbiome of Esslingen and Weiden (Figure 4A,C). These findings imply that the presence of human pathogenic species of Bb by feeding into the tick microbiome caused a shift and rearrangement of interactions within the tick microbiome. Thus, the natural tick microbiome is disturbed, reflected in more unspecific interactions between the bacteria and the lower number of clusters (Table 1).

Members of the genus *Rickettsia* were connectors in *Borrelia*-negative microbiomes (Figure 4A,C) but were peripherals in the *Borrelia*-positive microbiomes. Therefore, any acquirements of *Borrelia* species lead to a less prominent role of *Rickettsia* within the tick microbiome, indicating that not only the sequence read abundances were reduced, but also the bacterial interactions and functions were displaced. In addition, the members of the genus *Ochrobactrum* were characterized as connectors in the microbiomes from Esslingen (Figure 4A,B), which co-occurred with less mutual exclusions if *Rickettsia* were present in high sequence read abundances in EN (Supplementary Figure S17), but high mutual exclusions, if *Rickettsia* were present in very low sequence, read abundances in EP (Table 1, Supplementary Table S2 and Supplementary Figure S18). Thus, members of the genera *Rickettsia* and *Ochrobactrum* are facing strong competition once *Borrelia* was present. If *Borrelia* was absent, both genera had more freedom to each other, and required resources could be more likely shared. Reconstruction of *Rickettsia* genomes showed that all relevant genes for folic acid biosynthesis are present. The supply of folic acids is essential for the tick due to its unbalanced diet [71]. As the resource requirements for folic acid production in a substrate-limited tick environment [21] are demanding, the higher frequency of mutual exclusions of *Rickettsia* can be explained by bacterial competition for resources. These findings from Esslingen were in line with those from Weiden; however, networks from Weiden revealed fewer mutual exclusions (Supplementary Figures S19 and S20). Moreover, the WN network revealed an increase in mutual exclusions in the genus *Stenotrophomonas* compared to the EN network (Supplementary Figures S21–S24), which is most properly based on the higher sequence read abundances of *Stenotrophomonas* in Weiden (Figure 1B and Figure 3B), as well as the negative interactions (mutual exclusions) of *Ochrobactrum*.

Thus, it can be hypothesized that *Rickettsia* provides important defensive endosymbionts that protect the tick from *Borrelia* colonization [71]. If *Rickettsia* was absent in the tick microbiome, such protective function was likely to be carried out by members of the genus *Stenotrophomonas*, which is supported by the change in the topological role of *Stenotrophomonas* from a connector in WN to a network hub in WP.

## 5. Conclusions

The presence of human pathogenic Bb species significantly shifted the sequence read abundances and important topological roles of the tick microbiome. Moreover, the location was less important in the tick microbiome composition, and sequence read abundances were important in the location-dependent topological network role, indicating that location characteristics changed bacterial interaction patterns. Since infection with human pathogenic Bb species and other tick-associated pathogens varies regionally, we suggest that a bacterial 16S rRNA gene-based microbiome analysis should be included for both tick and host in the case of *Borrelia*-positive findings. The data foundation of a combined diagnostic approach of qPCR and amplicon sequencing will enable valid decisions for adequate treatment of human pathogenic Bb species and additional co-occurring pathogens, as they were location-specific.

## Figures and Tables

**Table 1:** Overview of interaction patterns of bacterial genera with important topological roles from the tick-borne *Borrelia*-negative (EN; WN) and *Borrelia*-positive (EP; WP) co-occurrence networks obtained from Esslingen (E) and Weiden (W), respectively. The EN, WN, EP, and WP networks were composed of 62, 56, 38, and 44 bacterial community assemblages, respectively.

	EN		EP		WN		WP	
Interacting Bacterial Genera	Mutual Exclusions	Co-Occurence	Mutual Exclusions	Co-Occurence	Mutual Exclusions	Co-Occurence	Mutual Exclusions	Co-Occurence
*Borrelia*	/	/	/	1	/	/	1	1
*Rickettsia*	106	0	2	0	25	3	8	0
*Mycobacterium*	13	36	14	5	8	29	1	11
*Phenylobacterium*	14	10	11	8	5	4	1	8
*Ralstonia*	5	5	15	5	12	11	2	18
*Burkholderia*	1	3	2	6	23	8	7	15
*Ochrobactrum*	6	12	56	6	1	23	7	3
*Legionella*	3	14	0	20	0	35	3	10
*Williamsia*	2	36	41	24	10	35	5	37
*Rhizobium*	0	37	31	19	2	13	20	8
*Pseudobutyrivibrio*	/	/	7	22	2	37	2	18
*Pseudomonas*	8	2	5	0	16	5	5	6
*Bdellovibrio*	1	6	/	/	7	11	10	16
*Pelomonas*	1	8	5	8	3	9	17	22
*Spiroplasma*	2	0	7	3	4	3	13	3
*Stenotrophomonas*	2	4	3	10	36	9	60	3
*Ca.* Neoehrlichia	2	1	5	4	9	2	25	6
*Rhodococcus*	0	32	4	27	4	22	30	41

## Data Availability

The bacterial 16S rRNA gene sequences for tick samples were deposited in the NCBI nucleotide sequence databases under accession PRJNA698232.

## Supplementary Materials

The following are available online at https://www.mdpi.com/article/10.3390/microorganisms9091814/s1, Figure S1: Mapped *Borrelia*-negative findings (A) and *Borrelia*-positive findings (B) on a map of Germany with identification of the districts and district-free cities for the year 2018. Colors of the legend mark the available tick nucleic acid extracts to the corresponding *Borrelia* finding in the figure, Figure S2: Correspondence analysis of bacterial community compositions (A) and euclidean distance matrix based on ward.D2 method (B) of *Borrelia*-negative ticks retrieved from Esslingen (EN; *n* = 62; orange circle) or Weiden (WN; *n* = 56, blue circle) and *Borrelia*-positive ticks retrieved from Esslingen (EP; *n* = 38; orange square) or Weiden (WP; *n* = 44; blue square). Bacterial community composition is based on genus level with binary data. The eigenvalues of both axes and standard errors are shown (A). For clusters a heights of 0.6 were chosen and denoted in dashed boxes (B), Figure S3: Euclidean distance matrix based on ward.D2 method on genus level with binary data of *Borrelia*-negative ticks retrieved from Esslingen (EN; *n* = 62) or Weiden (WN; *n* = 56) and *Borrelia*-positive ticks retrieved from Esslingen (EP; *n* = 38) or Weiden (WP; *n* = 44). For clusters a heights of 2 were chosen and denoted in colored boxes, Figure S4: Correspondence analysis of bacterial community compositions (A) and euclidean distance matrix based on ward.D2 method (B) of *Borrelia*-negative ticks retrieved from Esslingen (EN; *n* = 62; orange circle) or Weiden (WN; *n* = 56, blue circle) and *Borrelia*-positive ticks retrieved from Esslingen (EP; *n* = 38; orange square) or Weiden (WP; *n* = 44; blue square) after removal of the borrelia sequences. Bacterial community composition is based on genus level with relative abundances data. The eigenvalues of both axes and standard errors are shown (A). For clusters a heights of 0.6 were chosen and denoted in dashed boxes (B), Figure S5: *Borrelia*-negative network from Esslingen (EN; *n* = 62). Green lines indicate co-occurrence links, while red lines indicate mutual exclusion links. The different colors of the squares with the bacterial genera indicate different cluster membership, Figure S6: *Borrelia*-positive network from Esslingen (EP; *n* = 44). Green lines indicate co-occurrence links, while red lines indicate mutual exclusion links. The different colors of the squares with the bacterial genera indicate different cluster membership, Figure S7: *Borrelia*-negative network from Weiden (WN; *n* = 56). Green lines indicate co-occurrence links, while red lines indicate mutual exclusion links. The different colors of the squares with the bacterial genera indicate different cluster membership, Figure S8: *Borrelia*-positive network from Weiden (WP; *n* = 44). Green lines indicate co-occurrence links, while red lines indicate mutual exclusion links. The different colors of the squares with the bacterial genera indicate different cluster membership, Figure S9: Representation of the *Borrelia*-negative network by community cluster (GLay) from Esslingen (EN; *n* = 62). Green lines indicate co-occurrence links, while red lines indicate mutual exclusion links. The different colors of the squares with the bacterial genera indicate different cluster membership, Figure S10: Representation of the *Borrelia*-positive network by community cluster (GLay) from Esslingen (EP; *n* = 38). Green lines indicate co-occurrence links, while red lines indicate mutual exclusion links. The different colors of the squares with the bacterial genera indicate different cluster membership, Figure S11: Representation of the *Borrelia*-negative network by community cluster (GLay) from Weiden (WN; *n* = 56). Green lines indicate co-occurrence links, while red lines indicate mutual exclusion links. The different colors of the squares with the bacterial genera indicate different cluster membership, Figure S12: Representation of the *Borrelia*-positive network by community cluster (GLay) from Weiden (WP; *n* = 44). Green lines indicate co-occurrence links, while red lines indicate mutual exclusion links. The different colors of the squares with the bacterial genera indicate different cluster membership, Figure S13: Interaction of *Rickettsia* in the *Borrelia*-negative tick microbiome from Essling (EN; *n* = 62). Red lines indicate mutual exclusion links. The different colors of the squares with the bacterial genera indicate different cluster membership, Figure S14: Interaction of *Rickettsia* in the *Borrelia*-positive tick microbiome from Essling (EP; *n* = 62). Red lines indicate mutual exclusion links. The different colors of the squares with the bacterial genera indicate different cluster membership, Figure S15: Interaction of *Rickettsia* in the *Borrelia*-negative tick microbiome from Weiden (WN; *n* = 56). Green lines indicate co-occurrence links, while red lines indicate mutual exclusion links. The different colors of the squares with the bacterial genera indicate different cluster membership, Figure S16: Interaction of *Rickettsia* in the *Borrelia*-positive tick microbiome from Weiden (WP; *n* = 44). Green lines indicate co-occurrence links, while red lines indicate mutual exclusion links. The different colors of the squares with the bacterial genera indicate different cluster membership, Figure S17: Interaction of *Ochrobactrum* in the *Borrelia*-negative tick microbiome from Essling (EN; *n* = 62). Green lines indicate co-occurrence links, while red lines indicate mutual exclusion links. The different colors of the squares with the bacterial genera indicate different cluster membership, Figure S18: Interaction of *Ochrobactrum* in the *Borrelia*-positive tick microbiome from Essling (EP; *n* = 38). Green lines indicate co-occurrence links, while red lines indicate mutual exclusion links. The different colors of the squares with the bacterial genera indicate different cluster membership, Figure S19: Interaction of *Ochrobactrum* in the *Borrelia*-negative tick microbiome from Weiden (WN; *n* = 56). Green lines indicate co-occurrence links, while red lines indicate mutual exclusion links. The different colors of the squares with the bacterial genera indicate different cluster membership, Figure S20: Interaction of *Ochrobactrum* in the *Borrelia*-positive tick microbiome from Weiden (WP; *n* = 44). Green lines indicate co-occurrence links, while red lines indicate mutual exclusion links. The different colors of the squares with the bacterial genera indicate different cluster membership, Figure S21: Interaction of *Stenotrophomonas* in the *Borrelia*-negative tick microbiome from Essling (EN; *n* = 62). Green lines indicate co-occurrence links, while red lines indicate mutual exclusion links. The different colors of the squares with the bacterial genera indicate different cluster membership, Figure S22: Interaction of *Stenotrophomonas* in the *Borrelia*-positive tick microbiome from Essling (EP; *n* = 62). Green lines indicate co-occurrence links, while red lines indicate mutual exclusion links. The different colors of the squares with the bacterial genera indicate different cluster membership, Figure S23: Interaction of *Stenotrophomonas* in the *Borrelia*-negative tick microbiome from Weiden (WN; *n* = 56). Green lines indicate co-occurrence links, while red lines indicate mutual exclusion links. The different colors of the squares with the bacterial genera indicate different cluster membership, Figure S24: Interaction of *Stenotrophomonas* in the *Borrelia*-positive tick microbiome from Weiden (WP; *n* = 44). Green lines indicate co-occurrence links, while red lines indicate mutual exclusion links. The different colors of the squares with the bacterial genera indicate different cluster membership, Table S1: OTU count table, and OTU count table summarized on genus level, Table S2: OTU count table summarized on genus level with the relative sequence read abundance and topological role in the network of *Borrelia*-negative and -positive ticks from Esslingen and Weiden, respectively, Table S3: OTU diversity indices from the tick-borne *Borrelia*-negative (EN, *n* = 62; WN, *n* = 56) and *Borrelia*-positive microbiome (EP, *n* = 38; WP, *n* = 44) derived from Esslingen (E) or Weiden (W), Table S4: Parameters and topological roles from the tick-borne *Borrelia*-negative (EN; WN) and *Borrelia*-positive co-occurrence networks (EP; WP) derived from Esslingen (E) or Weiden (W). The EN, WN, EP and WP network consisted of 62, 56, 38 and 44 bacterial community compositions, respectively.
