# Supplementary material for: Presence of Human Pathogens of the Borrelia burgdorferi sensu lato Complex Shifts the Sequence Read Abundances of Tick Microbiomes in Two German Locations
Source: Microorganisms. 2021 Aug 26;9(9):1814. doi: 10.3390/microorganisms9091814 (PMC8469654; doi:10.3390/microorganisms9091814)
Supplement: Supplementary file 1 [file microorganisms-09-01814-s001.zip › microorganisms-1332188-supplementary.pdf]

Supplemental Information for:

Presence of Human Pathogens of the Borrelia burgdorferi sensu lato Complex Shifts the Sequence Read Abundances of Tick Microbiomes in Two German Locations

Angeline Hoffmann[1], Thomas Müller[2], Volker Fingerle[3] and Matthias Noll[1,4,*]

1 Department of Applied Sciences, Institute for Bioanalysis, Coburg University of Applied Sciences and Arts, 96450 Coburg, Germany; 2 Synlab Medical Care Unit, Department of Molecular Biology, Tick Laboratory, Weiden in the Upper Palati-nate, 92637 Weiden, Germany; 3 Bavarian Health and Food Safety Authority (LGL), National Reference Center for Borrelia, 85764 Oberschleißheim, Germany; 4 Bayreuth Center of Ecology and Environmental Research (BayCEER), University of Bayreuth, 95440Bayreuth, Germany

*** Correspondence: matthias.noll@hs-coburg.de; Tel.: +49-956-131-7645**

Table of contents:

Supplementary Figure 1: Mapped *Borrelia*-negative and *Borrelia*-positive findings on a map of Germany
Supplementary Figure 2: Grouped correspondence analysis and euclidean distance matrix with binary data
Supplementary Figure 3: Complete euclidean distance matrix with binary data
Supplementary Figure 4: Grouped correspondence analysis and euclidean distance matrix with relative abundances without *Borrelia* sequences
Supplementary Figure 5: *Borrelia*-negative network from Esslingen
Supplementary Figure 6: *Borrelia*-positive network from Esslingen
Supplementary Figure 7: *Borrelia*-negative network from Weiden
Supplementary Figure 8: *Borrelia*-positive network from Weiden
Supplementary Figure 9: Community cluster (GLay) of *Borrelia*-negative network from Esslingen
Supplementary Figure 10: Community cluster (GLay) of *Borrelia*-positive network from Esslingen
Supplementary Figure 11: Community cluster (GLay) of *Borrelia*-negative network from Weiden
Supplementary Figure 12: Community cluster (GLay) of *Borrelia*-positive network from Weiden
Supplementary Figure 13: Interaction of *Rickettsia* of *Borrelia*-negative network from Esslingen
Supplementary Figure 14: Interaction of *Rickettsia* of *Borrelia*-positive network from Esslingen
Supplementary Figure 15: Interaction of *Rickettsia* of *Borrelia*-negative network from Weiden
Supplementary Figure 16: Interaction of *Rickettsia* of *Borrelia*-positive network from Weiden
Supplementary Figure 17: Interaction of *Ochrobactrum* of *Borrelia*-negative network from Esslingen Supplementary Figure 18: Interaction of *Ochrobactrum* of *Borrelia*-positive network from Esslingen
Supplementary Figure 19: Interaction of *Ochrobactrum* of *Borrelia*-negative network from Weiden
Supplementary Figure 20: Interaction of *Ochrobactrum* of *Borrelia*-positive network from Weiden
Supplementary Figure 21: Interaction of *Stenotrophomonas* of *Borrelia*-negative network from Esslingen
Supplementary Figure 22: Interaction of *Stenotrophomonas* of *Borrelia*-positive network from Esslingen
Supplementary Figure 23: Interaction of *Stenotrophomonas* of *Borrelia*-negative network from Weiden
Supplementary Figure 24: Interaction of *Stenotrophomonas* of *Borrelia*-positive network from Weiden

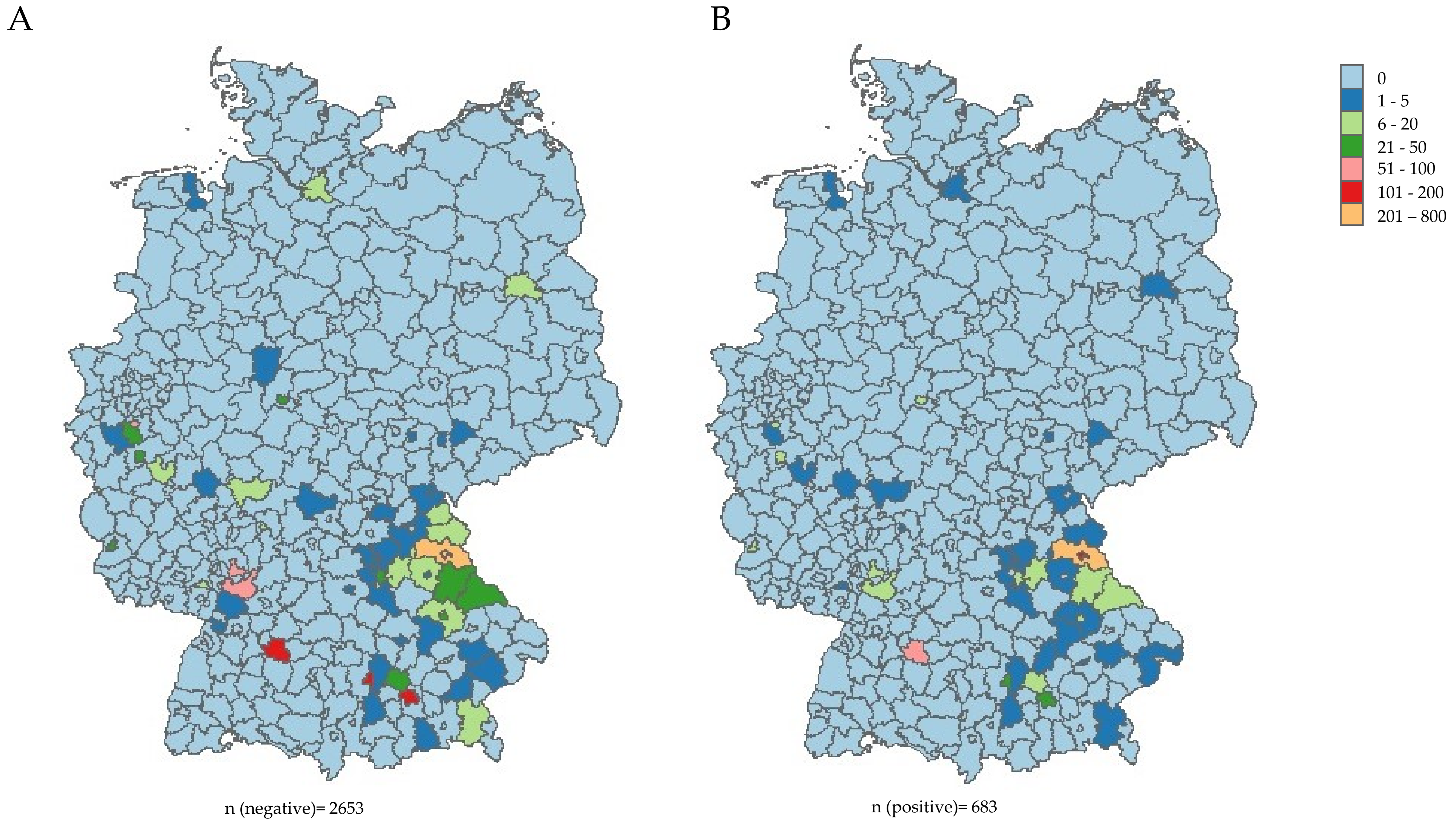

Figure S1. Mapped *Borrelia*-negative findings (A) and *Borrelia*-positive findings (B) on a map of Germany with identification of the districts and district-free cities for the year 2018. Colors of the legend mark the available tick nucleic acid extracts to the corresponding *Borrelia* finding in the figure.

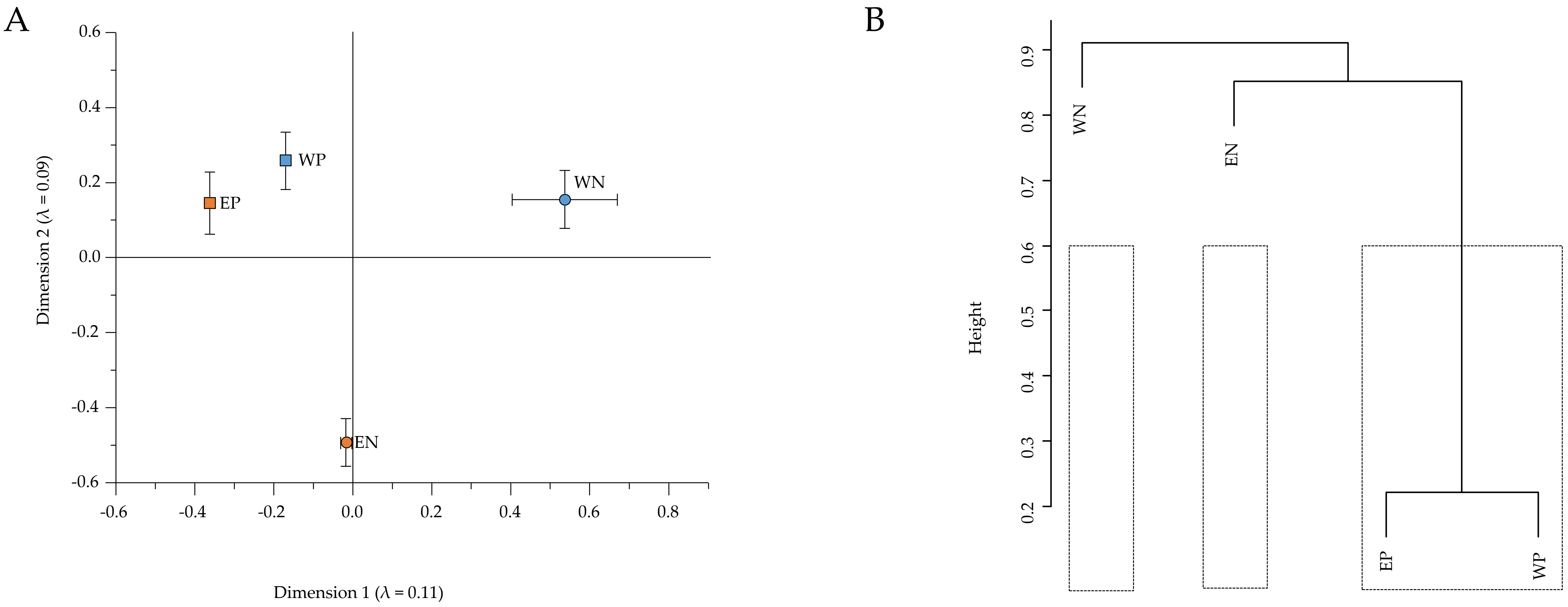

Figure S2. Correspondence analysis of bacterial community compositions (A) and euclidean distance matrix based on ward.D2 method (B) of *Borrelia*-negative ticks retrieved from Esslingen (EN; n=62; orange circle) or Weiden (WN; n=56, blue circle) and *Borrelia*-positive ticks retrieved from Esslingen (EP; n=38; orange square) or Weiden (WP; n=44; blue square). Bacterial community composition is based on genus level with binary data. The eigenvalues of both axes and SE are shown (A). For clusters a heights of 0.6 were chosen and denoted in dashed boxes (B).

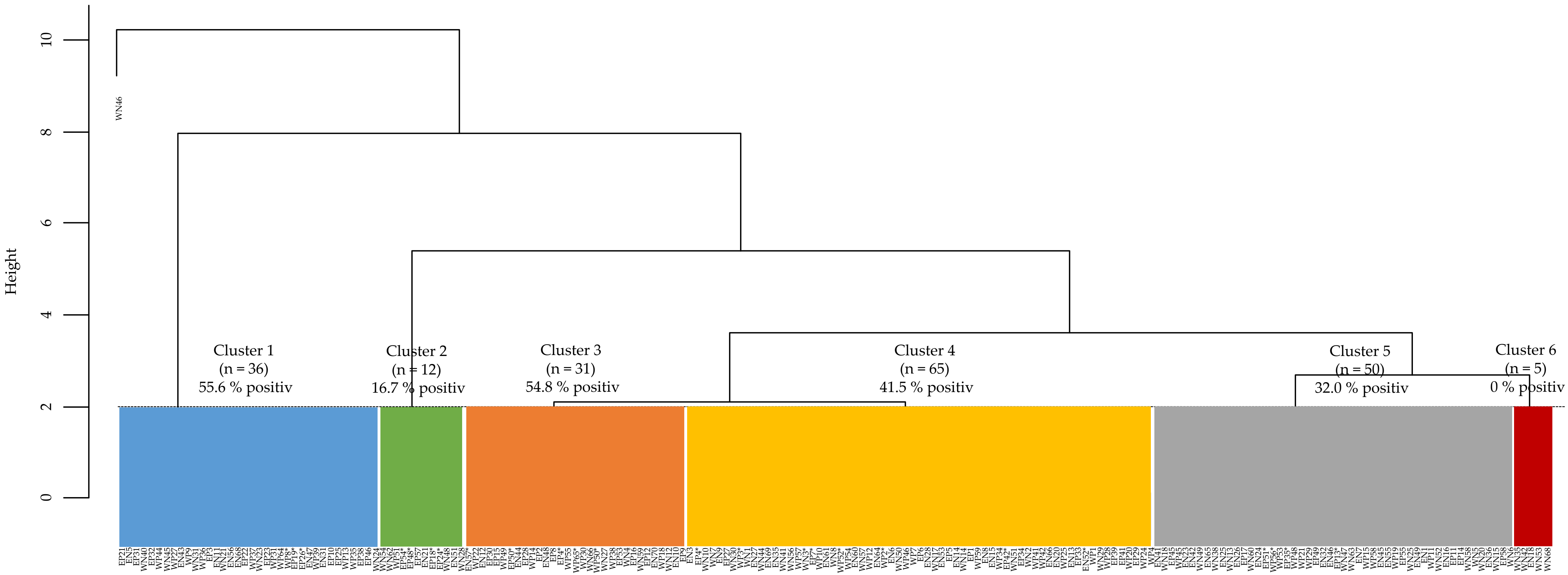

Figure S3. Euclidean distance matrix based on ward.D2 method on genus level with binary data of *Borrelia*-negative ticks retrieved from Esslingen (EN; n=62) or Weiden (WN; n=56) and *Borrelia*-positive ticks retrieved from Esslingen (EP; n=38) or Weiden (WP; n=44). For clusters a heights of 2 were chosen and denoted in colored boxes.

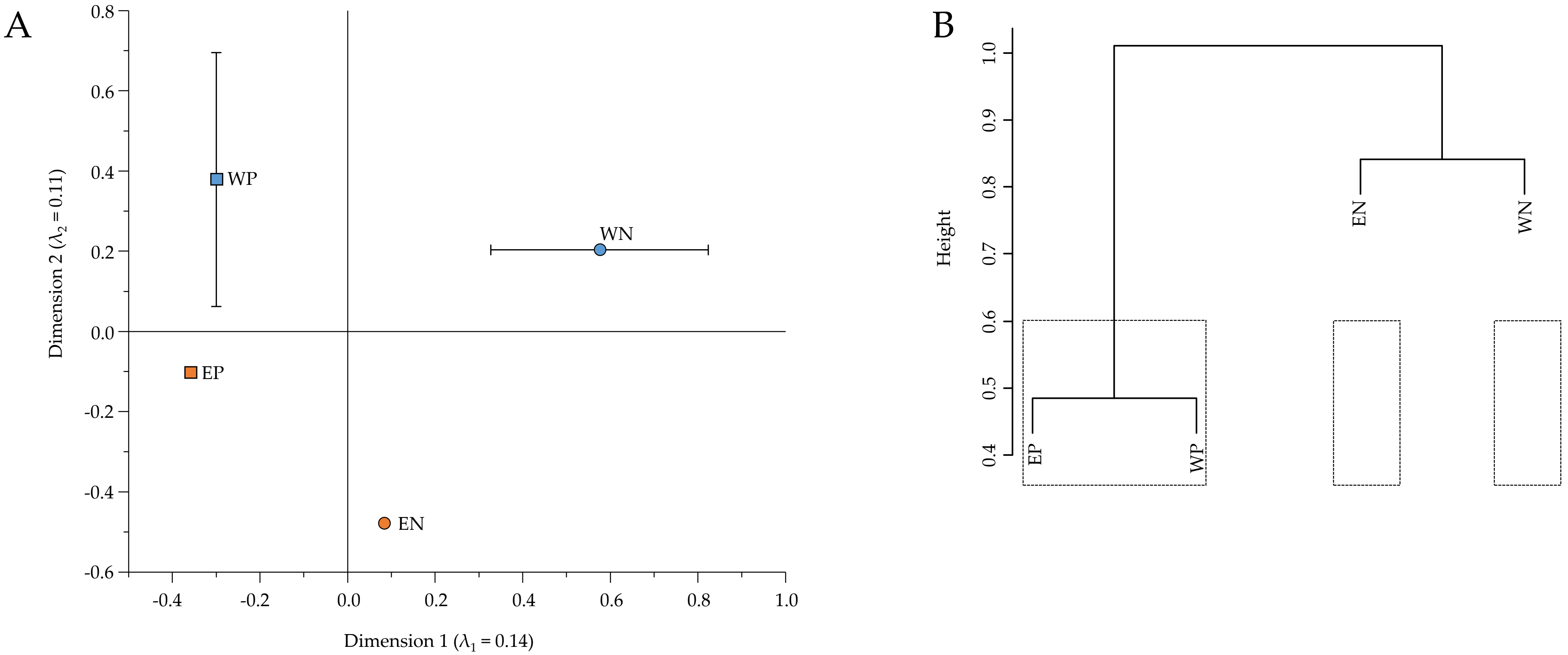

Figure S4. Correspondence analysis of bacterial community compositions (A) and euclidean distance matrix based on ward.D2 method (B) of *Borrelia*-negative ticks retrieved from Esslingen (EN; n=62; orange circle) or Weiden (WN; n=56, blue circle) and *Borrelia*-positive ticks retrieved from Esslingen (EP; n=38; orange square) or Weiden (WP; n=44; blue square) after removal of the borrelia sequences. Bacterial community composition is based on genus level with relative abundances data. The eigenvalues of both axes and SE are shown (A). For clusters a heights of 0.6 were chosen and denoted in dashed boxes (B).

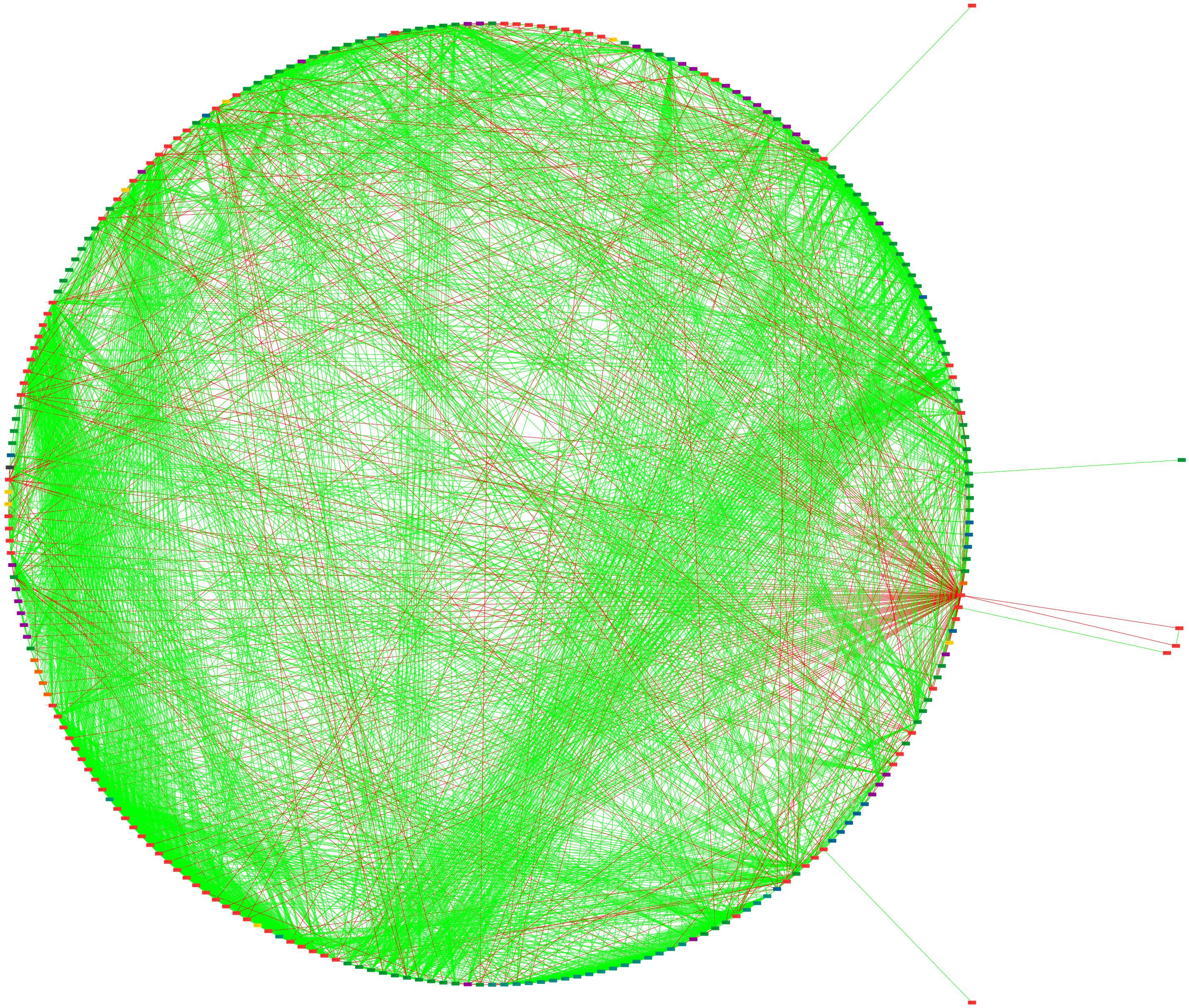

Figure S5. *Borrelia*-negative network from Esslingen (EN; n=62). Green lines indicate co-occurrence links, while red lines indicate mutual exclusion links. The different colors of the squares with the bacterial genera indicate different cluster membership.

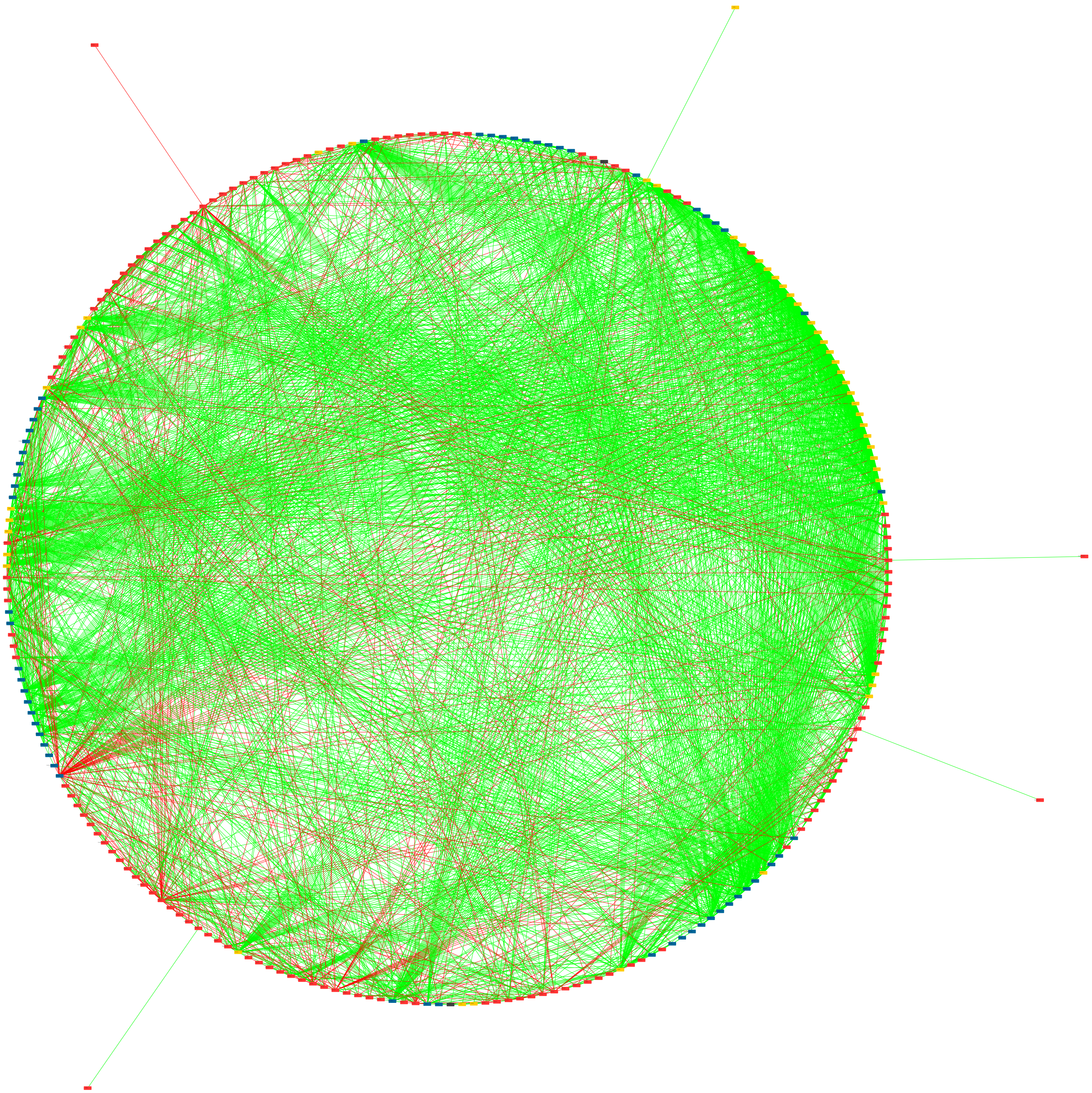

Figure S6. *Borrelia*-positive network from Esslingen (EP; n=44). Green lines indicate co-occurrence links, while red lines indicate mutual exclusion links. The different colors of the squares with the bacterial genera indicate different cluster membership.

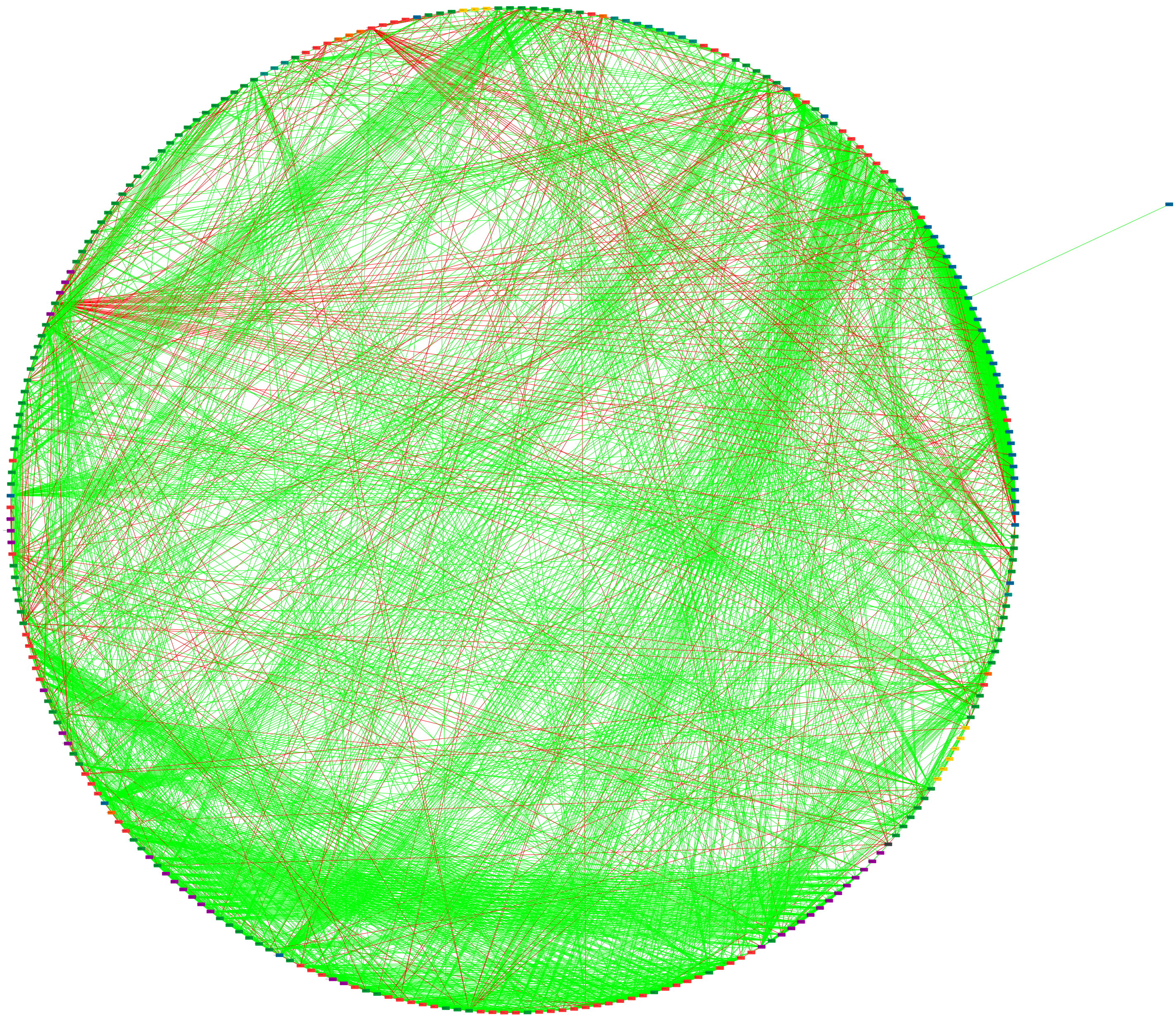

Figure S7. *Borrelia*-negative network from Weiden (WN; n=56). Green lines indicate co-occurrence links, while red lines indicate mutual exclusion links. The different colors of the squares with the bacterial genera indicate different cluster membership.

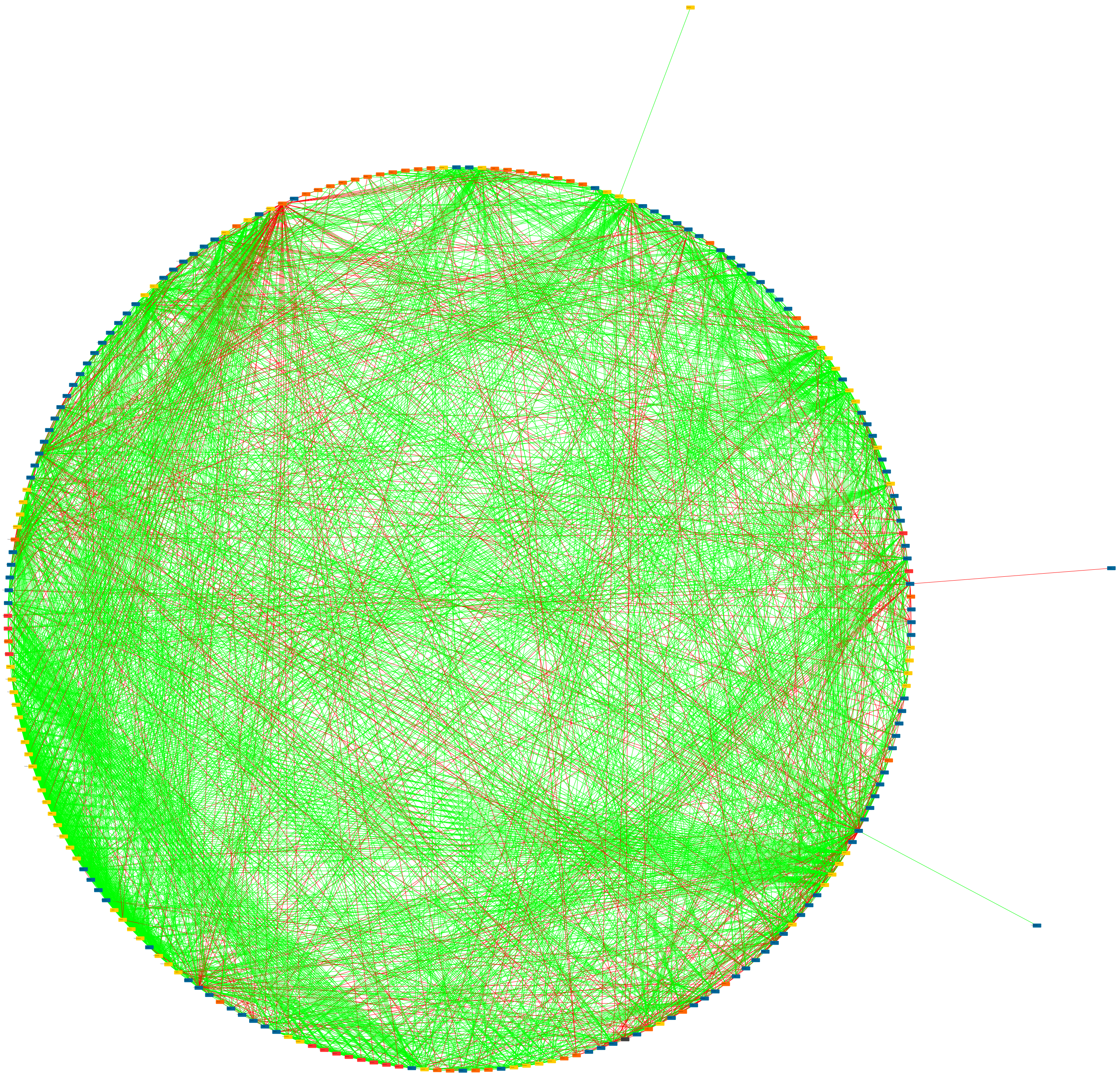

Figure S8. *Borrelia*-positive network from Weiden (WP; n=44). Green lines indicate co-occurrence links, while red lines indicate mutual exclusion links. The different colors of the squares with the bacterial genera indicate different cluster membership.

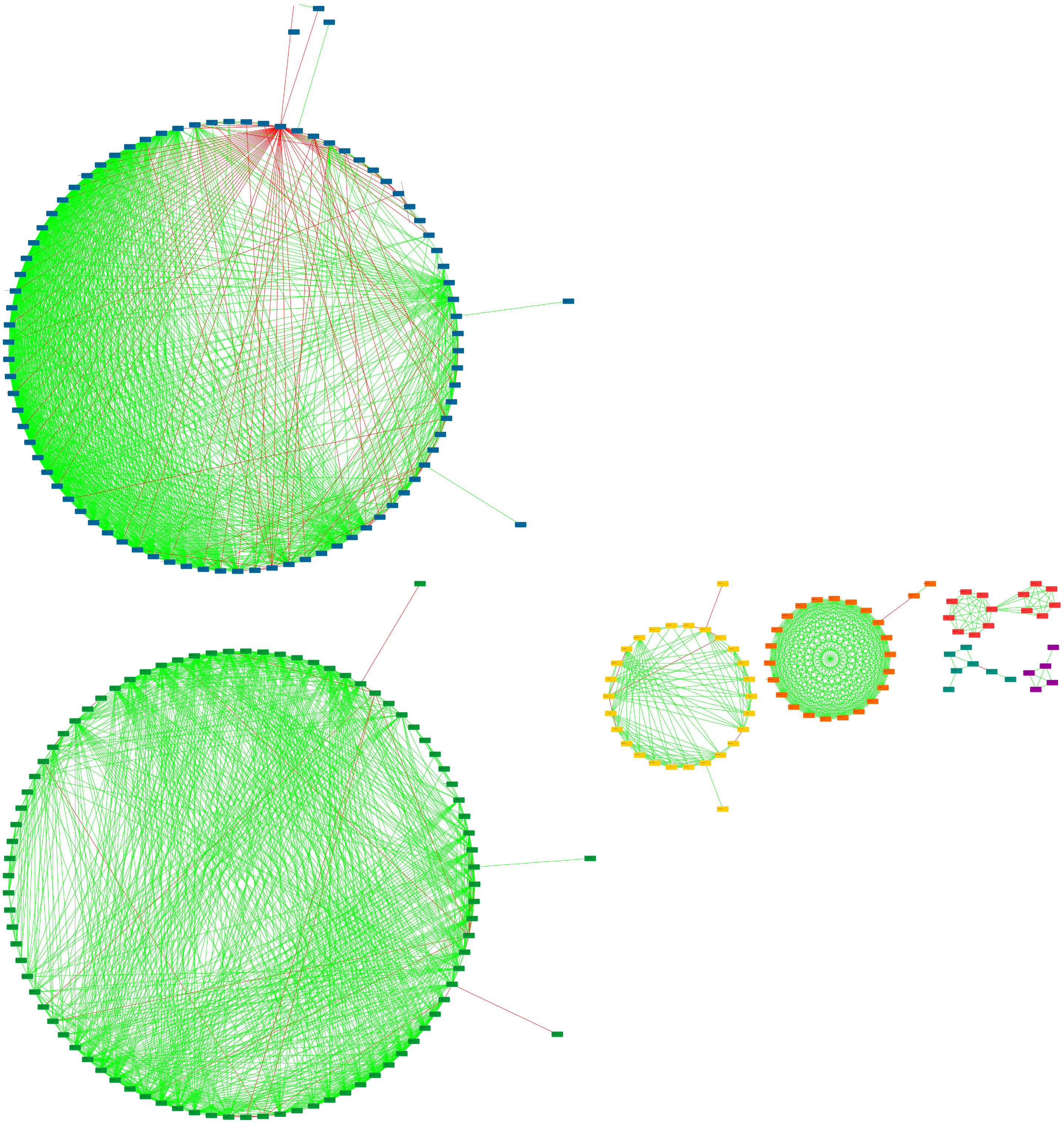

Figure S9. Representation of the *Borrelia*-negative network by community cluster (GLay) from Esslingen (EN; n=62). Green lines indicate co-occurrence links, while red lines indicate mutual exclusion links. The different colors of the squares with the bacterial genera indicate different cluster membership.

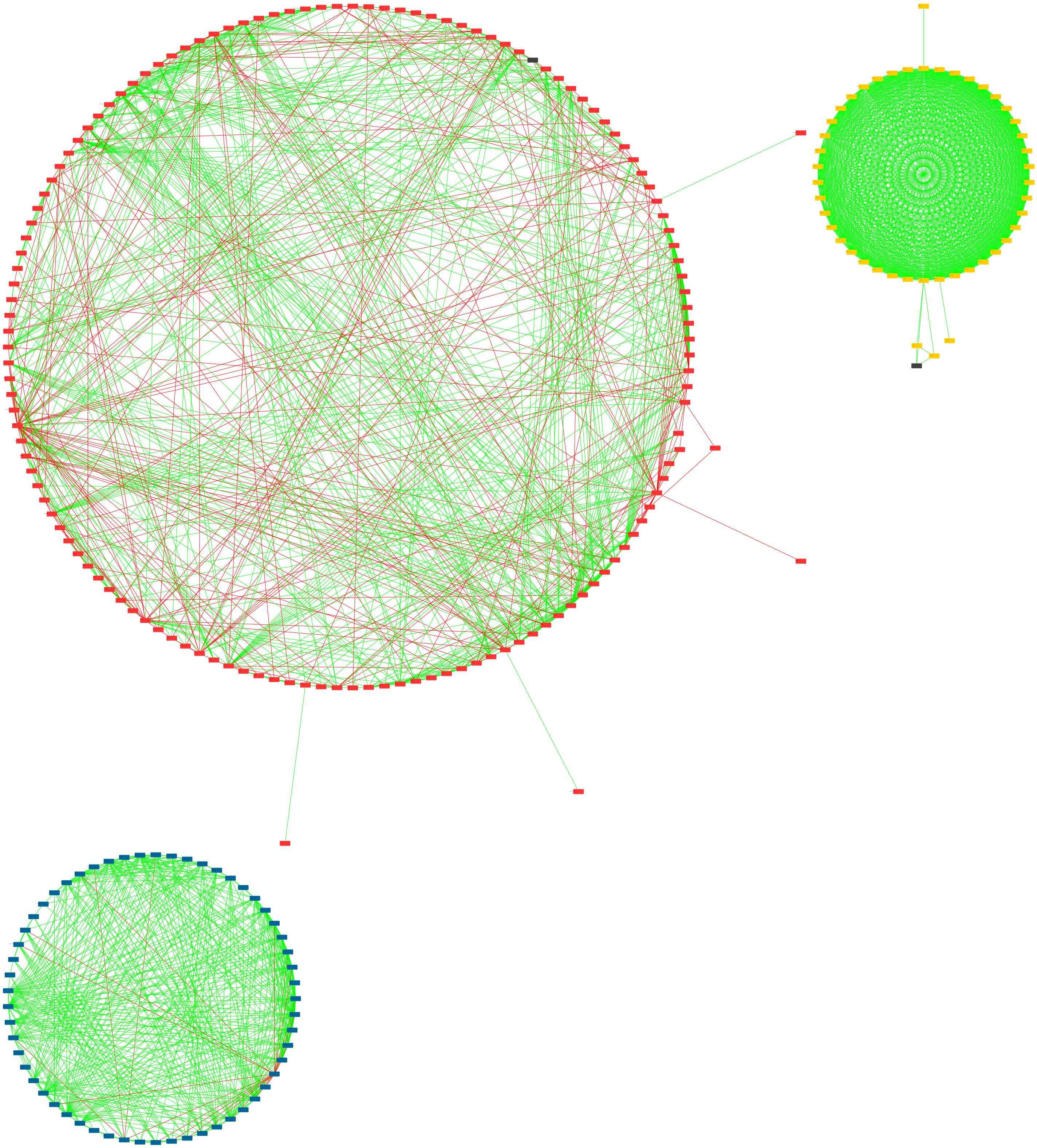

Figure S10. Representation of the *Borrelia*-positive network by community cluster (GLay) from Esslingen (EP; n=38). Green lines indicate co-occurrence links, while red lines indicate mutual exclusion links. The different colors of the squares with the bacterial genera indicate different cluster membership.

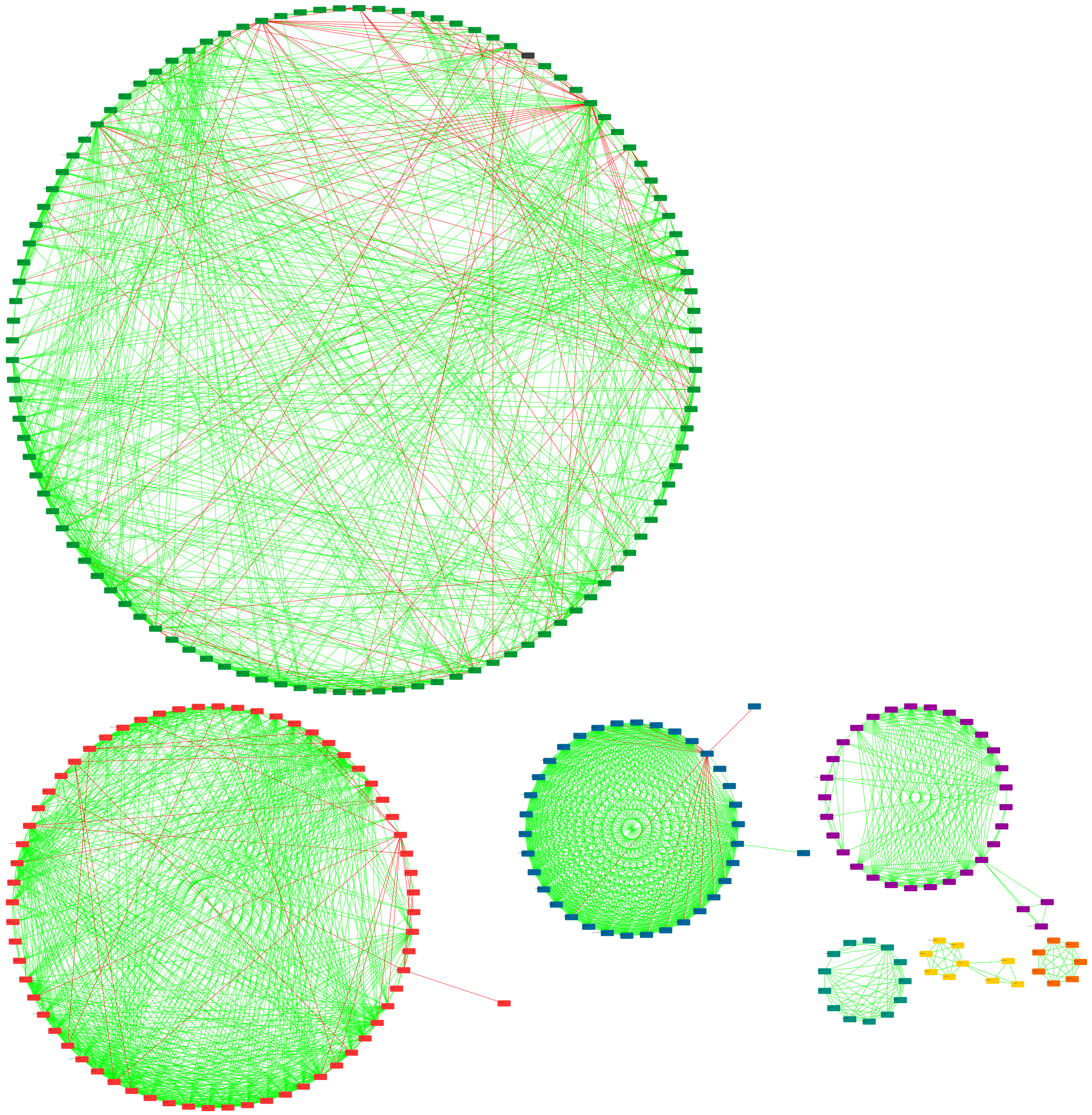

Figure S11. Representation of the *Borrelia*-negative network by community cluster (GLay) from Weiden (WN; n=56). Green lines indicate co-occurrence links, while red lines indicate mutual exclusion links. The different colors of the squares with the bacterial genera indicate different cluster membership.

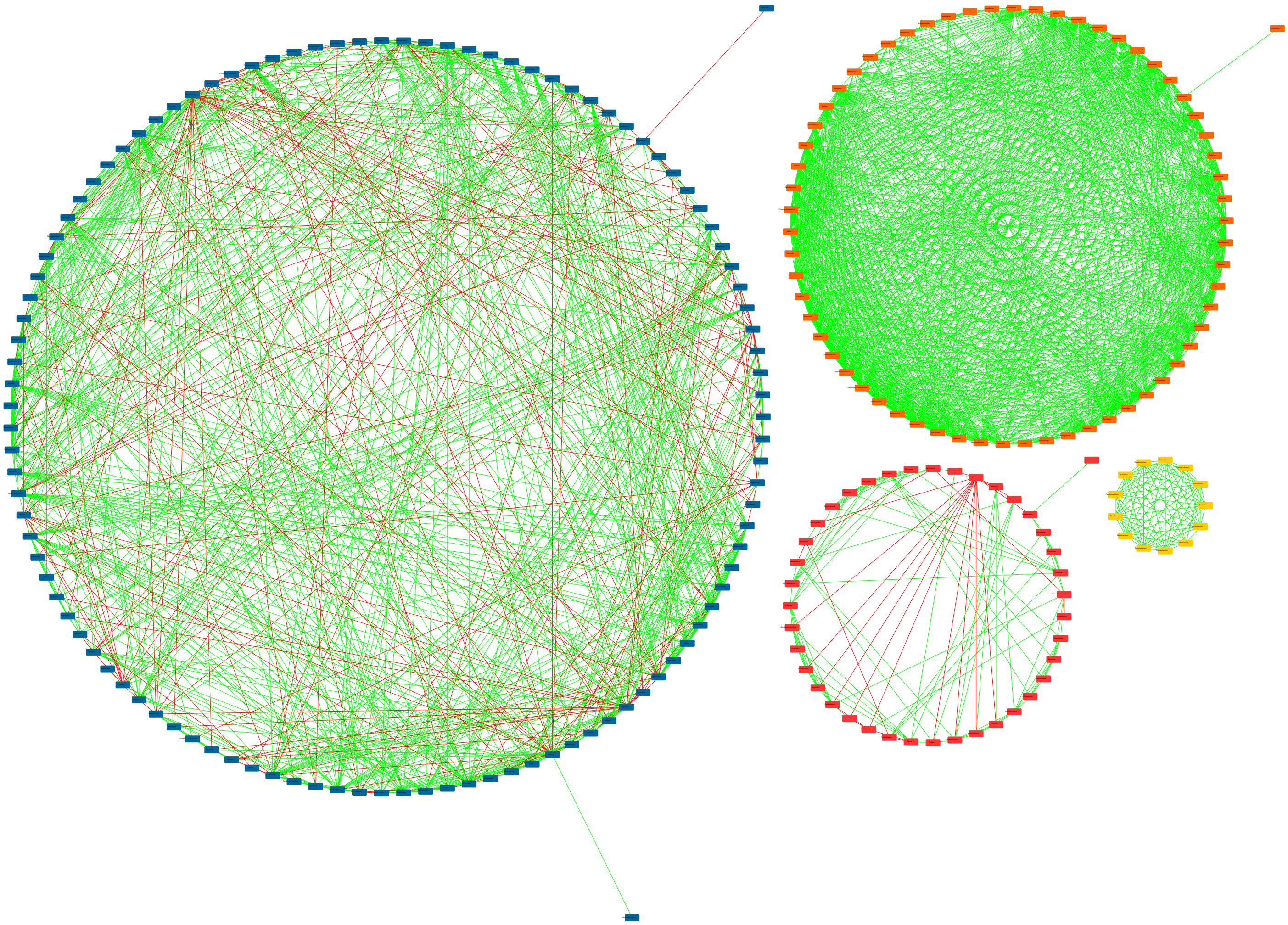

Figure S12. Representation of the *Borrelia*-positive network by community cluster (GLay) from Weiden (WP; n=44). Green lines indicate co-occurrence links, while red lines indicate mutual exclusion links. The different colors of the squares with the bacterial genera indicate different cluster membership.

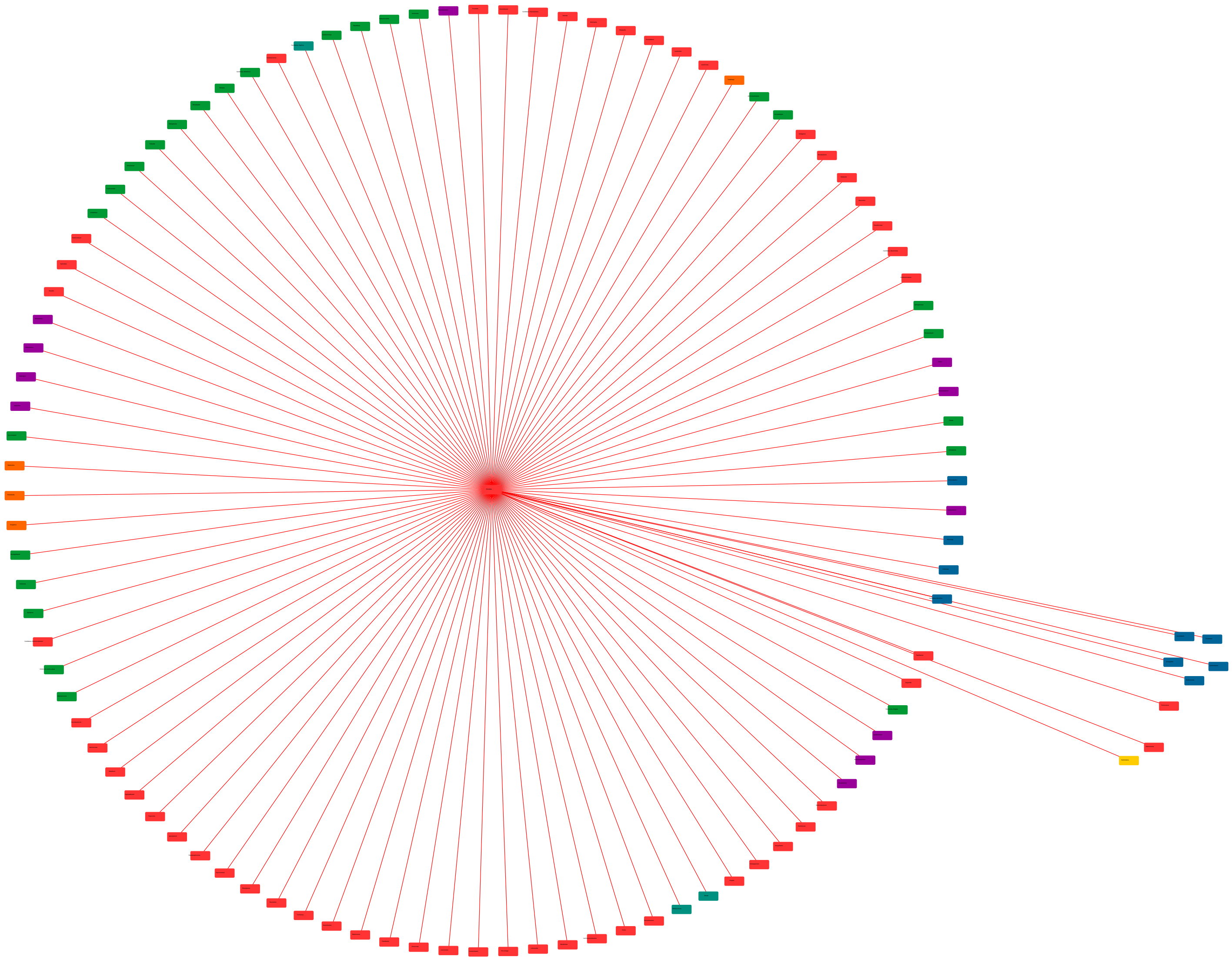

Figure S13. Interaction of *Rickettsia* in the *Borrelia*-negative tick microbiome from Esslingen (EN; n=62). Red lines indicate mutual exclusion links. The different colors of the squares with the bacterial genera indicate different cluster membership.

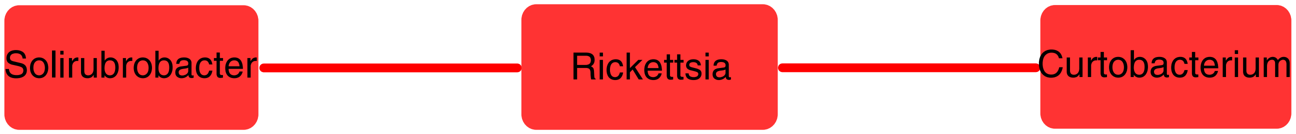

Figure S14. Interaction of *Rickettsia* in the *Borrelia*-positive tick microbiome from Esslingen (EP; n=62). Red lines indicate mutual exclusion links. The different colors of the squares with the bacterial genera indicate different cluster membership.

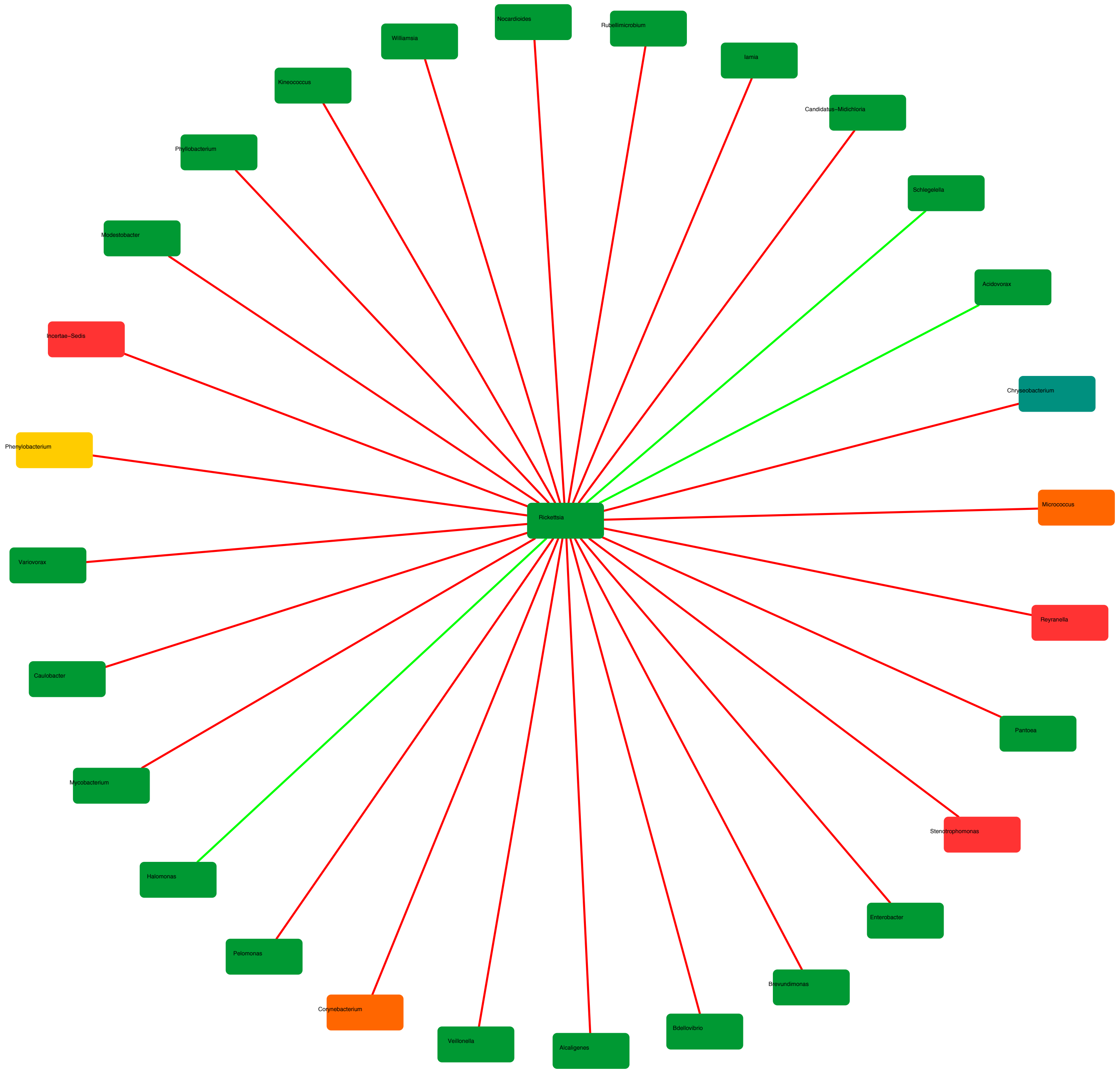

Figure S15. Interaction of *Rickettsia* in the *Borrelia*-negative tick microbiome from Weiden (WN; n=56). Green lines indicate co-occurrence links, while red lines indicate mutual exclusion links. The different colors of the squares with the bacterial genera indicate different cluster membership.

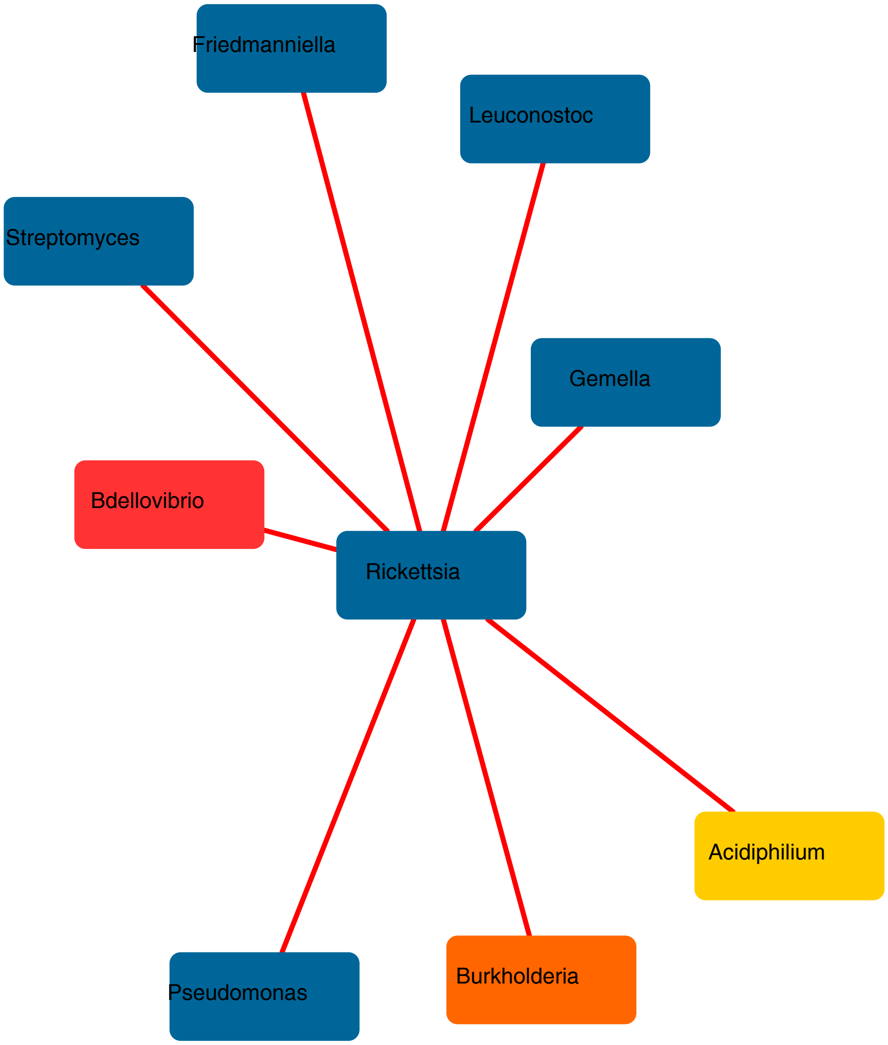

Figure S16. Interaction of *Rickettsia* in the *Borrelia*-positive tick microbiome from Weiden (WP; n=44). Green lines indicate co-occurrence links, while red lines indicate mutual exclusion links. The different colors of the squares with the bacterial genera indicate different cluster membership.

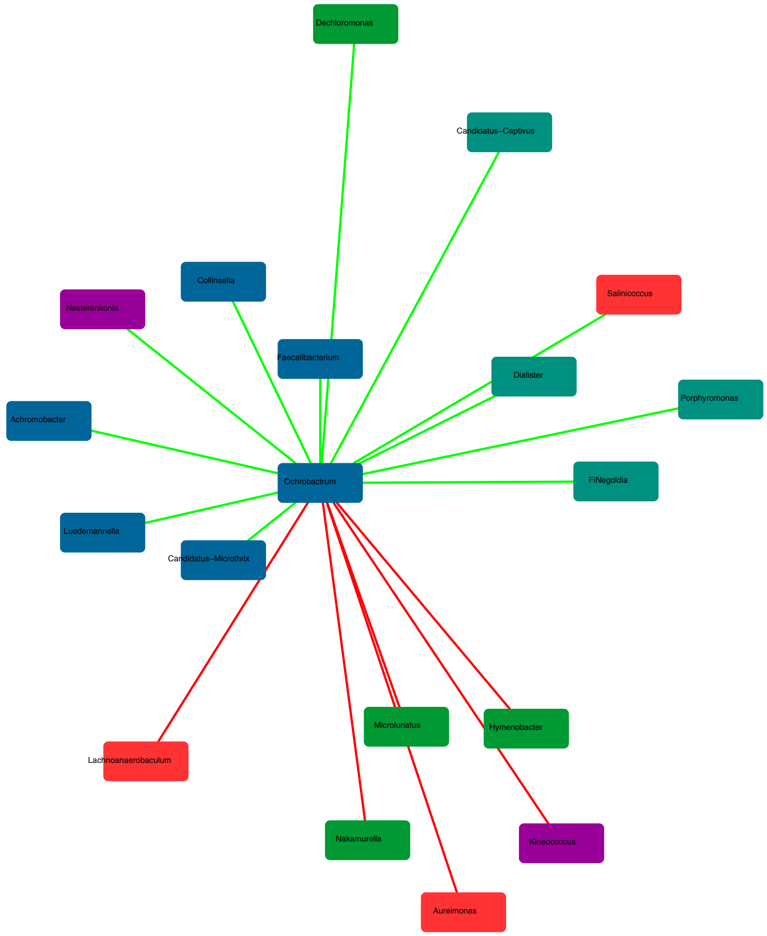

Figure S17. Interaction of *Ochrobactrum* in the *Borrelia*-negative tick microbiome from Esslingen (EN; n=62). Green lines indicate co-occurrence links, while red lines indicate mutual exclusion links. The different colors of the squares with the bacterial genera indicate different cluster membership.

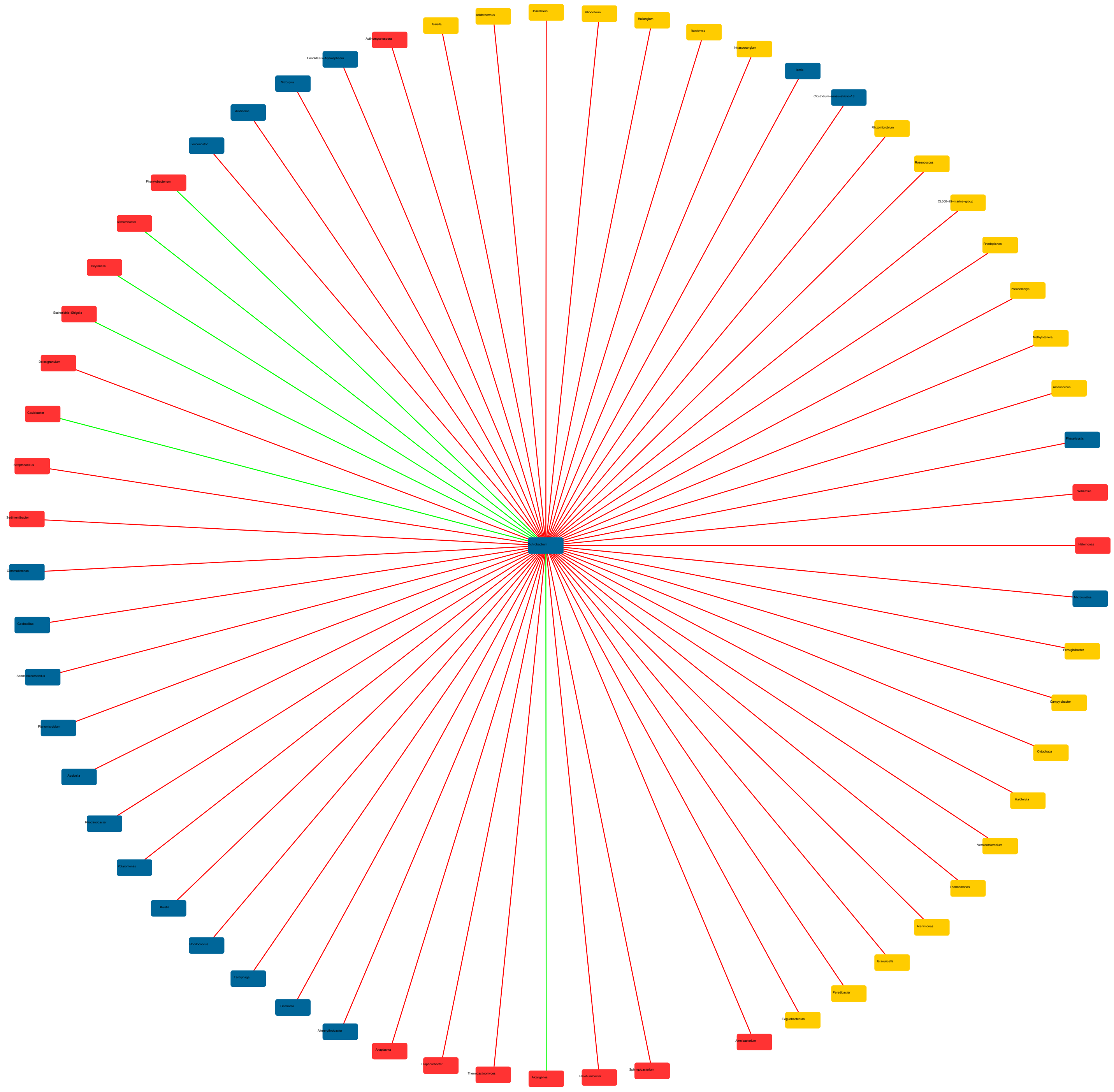

Figure S18. Interaction of *Ochrobactrum* in the *Borrelia*-positive tick microbiome from Esslingen (EP; n=38). Green lines indicate co-occurrence links, while red lines indicate mutual exclusion links. The different colors of the squares with the bacterial genera indicate different cluster membership.

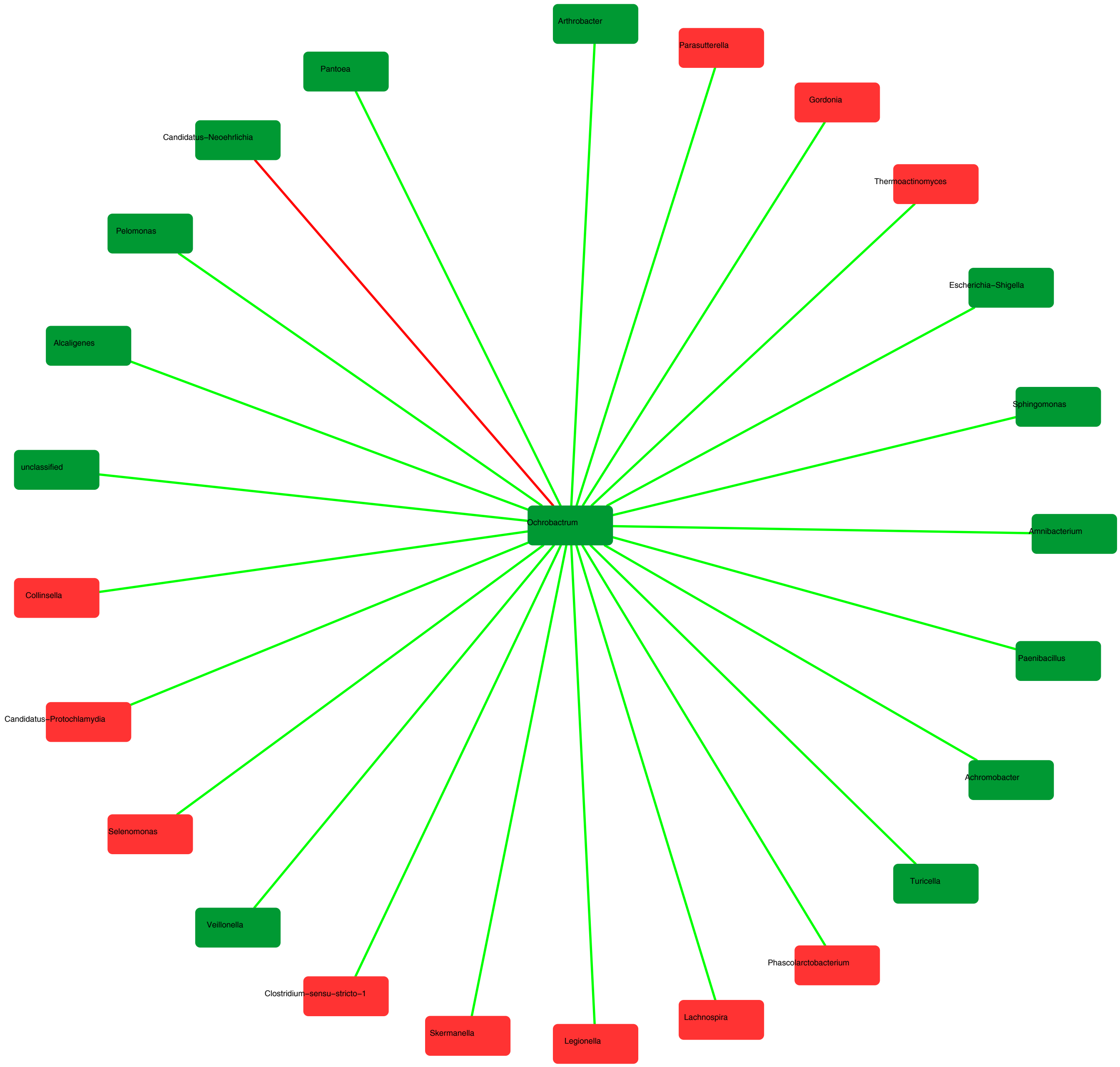

Figure S19. Interaction of *Ochrobactrum* in the *Borrelia*-negative tick microbiome from Weiden (WN; n=56). Green lines indicate co-occurrence links, while red lines indicate mutual exclusion links. The different colors of the squares with the bacterial genera indicate different cluster membership.

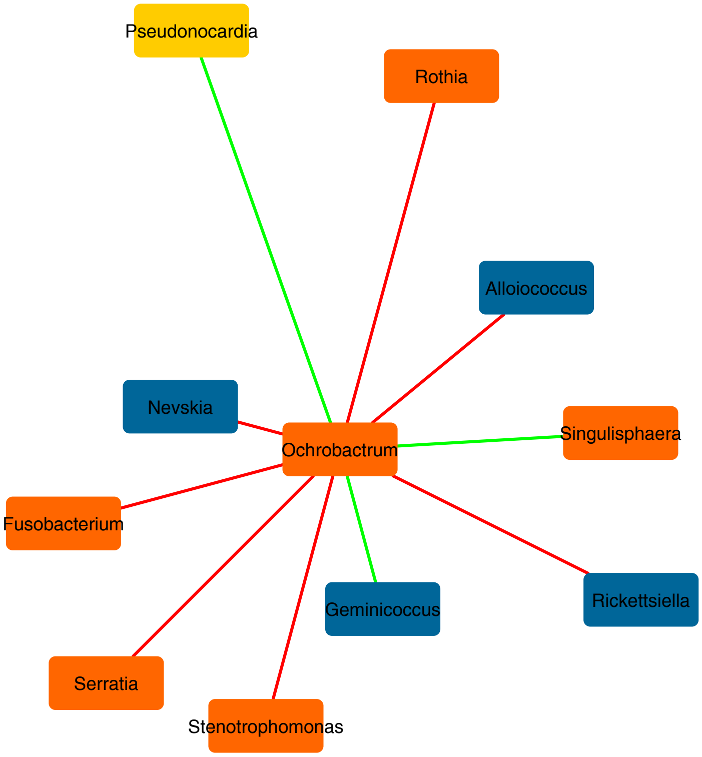

Figure S20. Interaction of *Ochrobactrum* in the *Borrelia*-positive tick microbiome from Weiden (WP; n=44). Green lines indicate co-occurrence links, while red lines indicate mutual exclusion links. The different colors of the squares with the bacterial genera indicate different cluster membership.

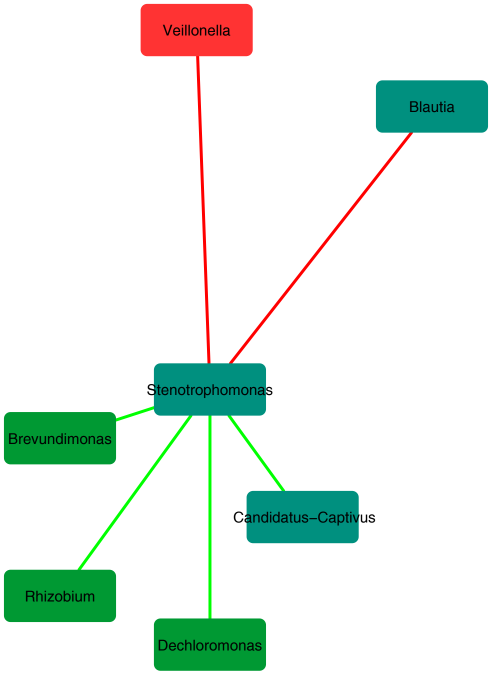

Figure S21. Interaction of *Stenotrophomonas* in the *Borrelia*-negative tick microbiome from Esslingen (EN; n=62). Green lines indicate co-occurrence links, while red lines indicate mutual exclusion links. The different colors of the squares with the bacterial genera indicate different cluster membership.

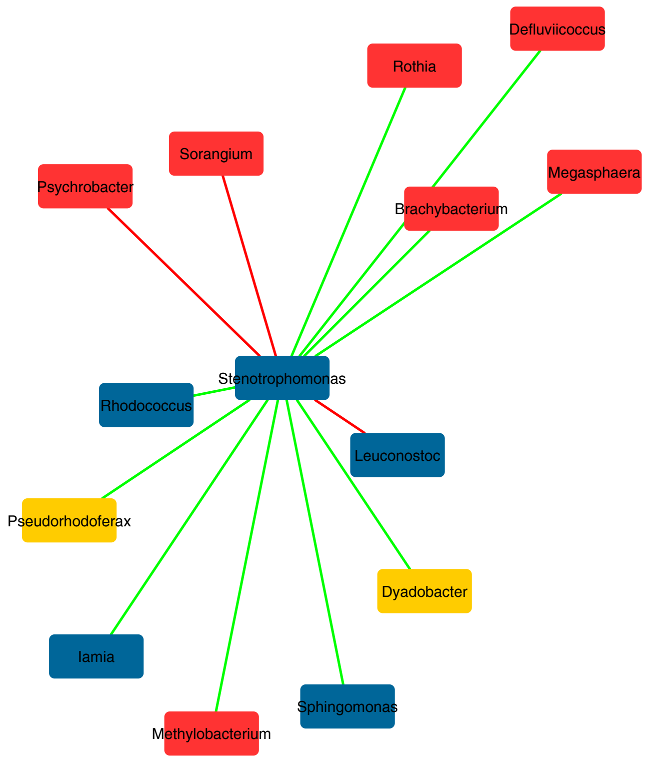

Figure S22. Interaction of *Stenotrophomonas* in the *Borrelia*-positive tick microbiome from Esslingen (EP; n=62). Green lines indicate co-occurrence links, while red lines indicate mutual exclusion links. The different colors of the squares with the bacterial genera indicate different cluster membership.

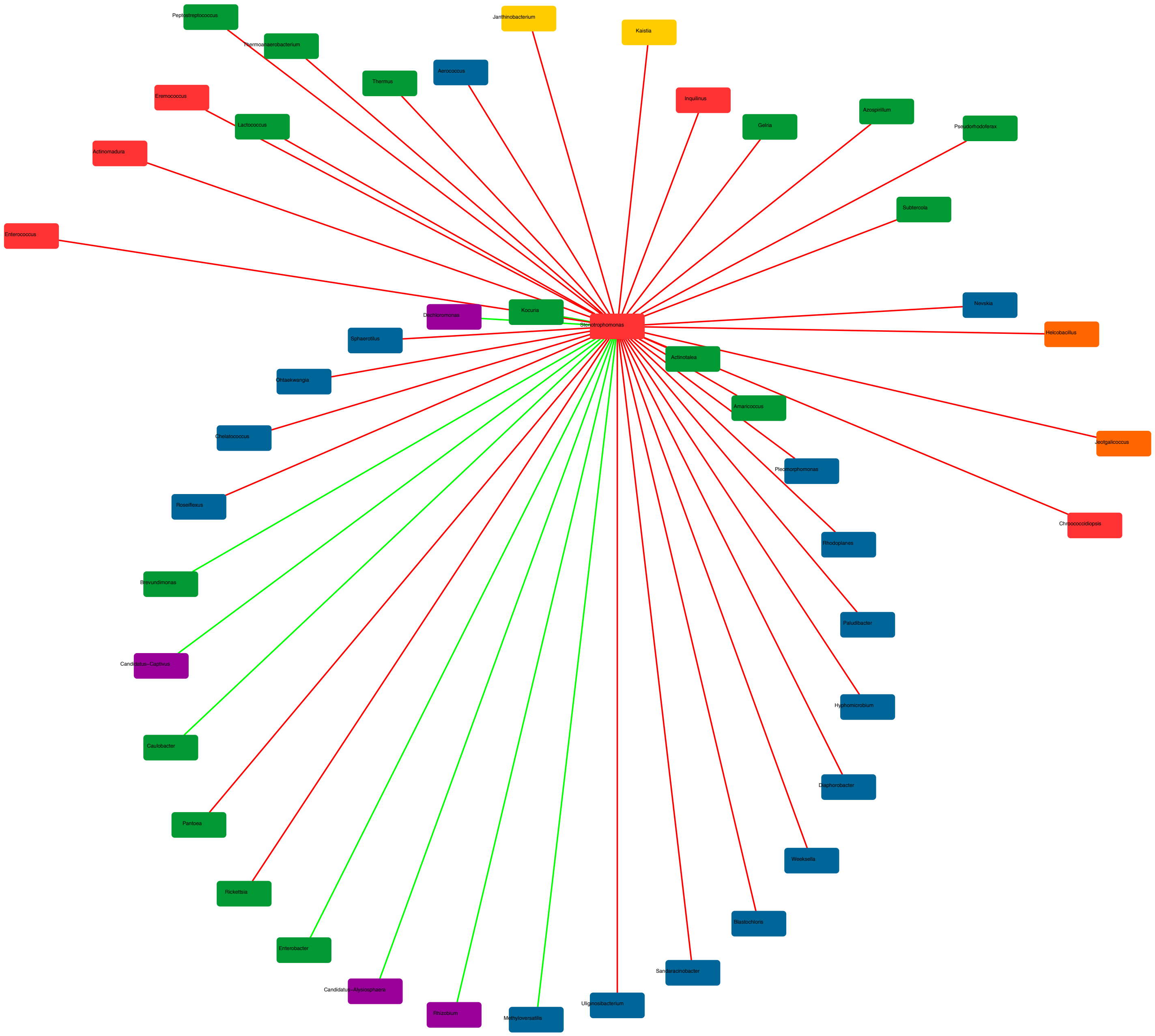

Figure S23. Interaction of *Stenotrophomonas* in the *Borrelia*-negative tick microbiome from Weiden (WN; n=56). Green lines indicate co-occurrence links, while red lines indicate mutual exclusion links. The different colors of the squares with the bacterial genera indicate different cluster membership.

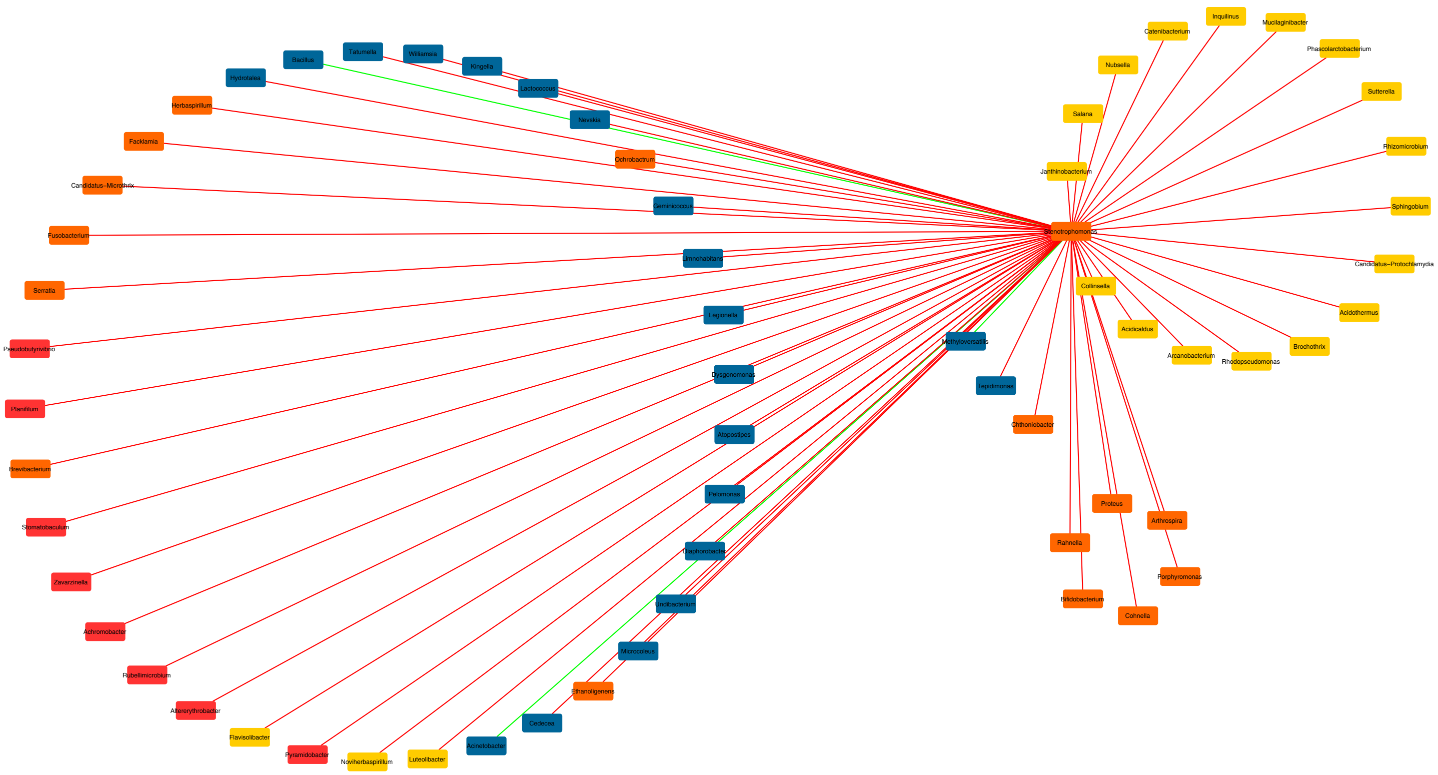

Figure S24. Interaction of *Stenotrophomonas* in the *Borrelia*-positive tick microbiome from Weiden (WP; n=44). Green lines indicate co-occurrence links, while red lines indicate mutual exclusion links. The different colors of the squares with the bacterial genera indicate different cluster membership.
